# Methylomic profiling of human brain tissue supports a neurodevelopmental origin for schizophrenia

**DOI:** 10.1186/s13059-014-0483-2

**Published:** 2014-10-28

**Authors:** Ruth Pidsley, Joana Viana, Eilis Hannon, Helen Spiers, Claire Troakes, Safa Al-Saraj, Naguib Mechawar, Gustavo Turecki, Leonard C Schalkwyk, Nicholas J Bray, Jonathan Mill

**Affiliations:** Institute of Psychiatry, King’s College London, London, SE5 8AF UK; Garvan Institute of Medical Research, Sydney, NSW 2010 Australia; University of Exeter Medical School, University of Exeter, Exeter, UK, RILD Building, Royal Devon & Exeter Hospital, Barrack Road, Exeter, EX2 5DW UK; Douglas Mental Health Institute, McGill University, Montreal, QC H4H 1R3 Canada; School of Biological Sciences, University of Essex, Colchester, CO4 3SQ UK

## Abstract

**Background:**

Schizophrenia is a severe neuropsychiatric disorder that is hypothesized to result from disturbances in early brain development. There is mounting evidence to support a role for developmentally regulated epigenetic variation in the molecular etiology of the disorder. Here, we describe a systematic study of schizophrenia-associated methylomic variation in the adult brain and its relationship to changes in DNA methylation across human fetal brain development.

**Results:**

We profile methylomic variation in matched prefrontal cortex and cerebellum brain tissue from schizophrenia patients and controls, identifying disease-associated differential DNA methylation at multiple loci, particularly in the prefrontal cortex, and confirming these differences in an independent set of adult brain samples. Our data reveal discrete modules of co-methylated loci associated with schizophrenia that are enriched for genes involved in neurodevelopmental processes and include loci implicated by genetic studies of the disorder. Methylomic data from human fetal cortex samples, spanning 23 to 184 days post-conception, indicates that schizophrenia-associated differentially methylated positions are significantly enriched for loci at which DNA methylation is dynamically altered during human fetal brain development.

**Conclusions:**

Our data support the hypothesis that schizophrenia has an important early neurodevelopmental component, and suggest that epigenetic mechanisms may mediate these effects.

**Electronic supplementary material:**

The online version of this article (doi:10.1186/s13059-014-0483-2) contains supplementary material, which is available to authorized users.

## Background

Schizophrenia is a severe neuropsychiatric disorder characterized by episodic psychosis and altered cognitive function. With a lifetime prevalence of approximately 1%, schizophrenia contributes significantly to the global burden of disease [1]. Although schizophrenia does not typically manifest until late adolescence or early adulthood, evidence from neuroimaging, neuropathology and epidemiological studies has led to its conceptualization as a neurodevelopmental disorder, with etiological origins before birth [2]. To date, however, the neurobiological mechanisms underlying the disorder remain largely undefined, and molecular evidence for *in utero* disturbances in schizophrenia is currently lacking.

Schizophrenia is known to have a substantial genetic component, involving a large number of common variants with individually small effects on risk for the disorder [3], as well as rarer mutations [4] and copy number variants [5] of greater effect size. Of note, several of the most robustly supported schizophrenia susceptibility genes have known roles in early brain development and appear to impact on schizophrenia risk during this period [6,7]. Epidemiological research suggests that prenatal environmental insults are also important, with established associations between hypoxia [8], maternal infection [9], maternal stress [10], and maternal malnutrition or famine [11] and risk for developing schizophrenia. These observations have led to a growing interest in the role of developmentally regulated epigenetic variation in the molecular etiology of schizophrenia [12]. The notion that epigenetic processes are involved in the onset of schizophrenia is supported by recent methylomic studies of disease-discordant monozygotic twins [13], clinical sample cohorts [14], and post-mortem brain tissue [15].

Here, we describe a systematic study of schizophrenia-associated methylomic variation in the adult brain and its relationship to changes in DNA methylation during human fetal brain development. We profiled DNA methylation in matched prefrontal cortex (PFC) and cerebellum brain tissue from schizophrenia patients and controls, subsequently assessing disease-associated regions in human fetal cortex samples spanning 23 to 184 days post-conception. Our data support the hypothesis that schizophrenia has an important early neurodevelopmental component, and suggest that epigenetic mechanisms likely contribute to these disturbances.

## Results and discussion

### Identification of schizophrenia-associated differentially methylated positions in the prefrontal cortex

**Figure 1:**
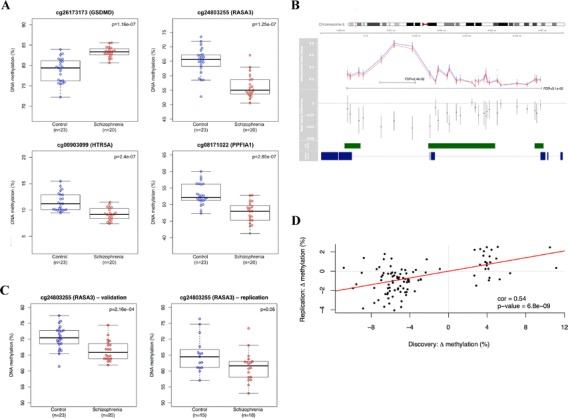
**Schizophrenia-associated DNA methylation differences in the prefrontal cortex. (A)** Probes associated with four genes are significantly associated with schizophrenia at FDR ≤0.05: *GSDMD* (control = 78.9 ± 3.0, schizophrenia = 83.3 ± 1.3, FDR = 0.03); *RASA3* (control = 65.1 ± 4.7, schizophrenia = 56.4 ± 4.1, FDR = 0.03); *HTR5A* (control = 11.5 ± 1.7, schizophrenia = 9.2 ± 1.2, FDR = 0.03); and *PPFIA1* (control = 53.1 ± 3.2, schizophrenia = 47.6 ± 3.2, FDR = 0.03). **(B)** The top-ranked differentially methylated region is located in an intergenic CpG island in *NRN1* where there is coordinated hypomethylation across 29 adjacent CpG sites in schizophrenia patients (red) compared with controls (blue). The mean DNA methylation difference across all 29 sites is -1.77% (FDR = 3.1e-2), with a notable region of hypomethylation (-3.7%) spanning three consecutive DMPs (FDR = 2.4e-2). **(C)** Selected schizophrenia-associated DMPs were successfully validated and replicated. Shown are technical validation data for the CpG site corresponding to probe cg24803255 in *RASA3* confirming significant schizophrenia-associated hypomethylation (control = 70.5 ± 3.7, schizophrenia = 66.4 ± 3.2, *P* = 0.0002). Additional technical validation data for other loci are shown in Figure S2 in Additional file 1. Also shown are replication data for cg24803255 in *RASA3* in PFC from the Montreal cohort confirming significant schizophrenia-associated hypomethylation (control = 65.1 ± 5.5, schizophrenia = 61.3 ± 4.7, *P* = 0.05). **(D)** DNA methylation differences at the 100 top-ranked PFC DMPs identified in the London discovery cohort are significantly correlated with schizophrenia-associated differences at the same probes in PFC from the Montreal replication cohort (r = 0.54, *P* = 6.8e-09).

Our ‘discovery’ cohort comprised PFC and cerebellum samples from schizophrenia patients and matched (for sex, age and sample quality markers (for example, pH)) control donors archived in the MRC London Brain Bank for Neurodegenerative Diseases (LBBND; see Materials and methods; Tables S1 and S2 in Additional file 1). Genome-wide patterns of DNA methylation were quantified using the Illumina Infinium HumanMethylation450 BeadChip (450K array) (Illumina Inc., San Diego, CA, USA), performing pre-processing, normalization and stringent quality control as previously described [16] (see Materials and methods; Table S3 in Additional file 1). In total, data from 43 PFC (20 schizophrenia and 23 controls) and 44 cerebellum (21 schizophrenia and 23 controls) samples passed quality control metrics and were used for analysis. The top-ranked differentially methylated positions (DMPs) in each brain region are shown in Tables S4 and S5 in Additional file 1. Most notably, highly significant DMPs were identified in the PFC (Table 1), with probes in four genes being significantly associated with schizophrenia at a false discovery rate (FDR) ≤0.05: *GSDMD* (cg26173173: control = 78.9 ± 3.0, schizophrenia = 83.3 ± 1.3, FDR = 0.03); *RASA3* (cg24803255: control = 65.1 ± 4.7, schizophrenia = 56.4 ± 4.1, FDR = 0.03); *HTR5A* (cg00903099: control = 11.5 ± 1.7, schizophrenia = 9.2 ± 1.2, FDR = 0.03); and *PPFIA1* (cg08171022: control = 53.1 ± 3.2, schizophrenia = 47.6 ± 3.2, FDR = 0.03) (Figure 1A). As epigenetic epidemiological research can be confounded by cellular heterogeneity [17,18], we used an *in silico* algorithm to assess the extent to which DMPs are influenced by differences in neuronal proportions between individuals [19]. Notably, all top-ranked PFC DMPs remained significantly associated with schizophrenia after correction for the estimated neuronal proportion in each sample (Table S4 in Additional file 1). In contrast to the PFC, few schizophrenia-associated DMPs (none at FDR ≤0.05) were identified in the cerebellum (Table S5 in Additional file 1), suggesting that disease-associated epigenetic differences are likely to be brain region-specific.

**Table 1:** **Schizophrenia-associated differentially methylated positions in the prefrontal cortex**

**Probe ID**	**Genomic position (hg19)**	**Gene**	**Gene region**	***P*** **-value**	**FDR**	**Mean SZ**	**Mean control**	**Beta difference**
cg26173173	chr8:144642813	*GSDMD*	Body	1.16E-07	0.03	0.83	0.79	0.04
cg24803255	chr13:114807060	*RASA3*	Body	1.25E-07	0.03	0.56	0.65	−0.09
cg00903099	chr7:154862441	*HTR5A*	TSS200	2.40E-07	0.03	0.09	0.12	−0.02
cg08171022	chr11:70185278	*PPFIA1*	Body	2.85E-07	0.03	0.48	0.53	−0.05
cg02857643	chr17:48696108	*CACNA1G*	Body	1.63E-06	0.1	0.87	0.92	−0.05
cg00236305	chr2:1796076	*MYT1L*	Body	1.75E-06	0.1	0.67	0.73	−0.06
cg14966346	chr14:104152124	*KLC1*	Body	2.51E-06	0.1	0.72	0.77	−0.05
cg13079528	chr7:4027346	*SDK1*	Body	2.75E-06	0.1	0.71	0.79	−0.08
cg14429765	chr8:6492531	*MCPH1*	Body	2.79E-06	0.1	0.79	0.76	0.03
cg08602214	chr8:22864392	*RHOBTB2*	Body	2.87E-06	0.1	0.69	0.75	−0.07
cg19735533	chr2:241196887	*-*	-	2.94E-06	0.1	0.76	0.81	−0.06
cg09507608	chr22:29873338	*-*	-	2.95E-06	0.1	0.56	0.62	−0.06
cg23844013	chr1:57383752	*C8A*	3' UTR	3.20E-06	0.1	0.82	0.79	0.03
cg26578910	chr19:47204096	*PRKD2*	Body	3.47E-06	0.1	0.67	0.71	−0.04
cg21847368	chr13:113744010	*MCF2L*	Body	3.70E-06	0.1	0.80	0.84	−0.03
cg03607729	chr16:25138698	*LCMT1*	Body	4.19E-06	0.1	0.70	0.75	−0.04
cg10248981	chr10:126157948	*LHPP*	Body	4.32E-06	0.1	0.55	0.63	−0.07
cg03445663	chr2:242170236	*HDLBP*	Body	4.59E-06	0.1	0.51	0.59	−0.08
cg15079231	chr3:42572745	*VIPR1*	Body	4.93E-06	0.1	0.76	0.80	−0.04
cg21341878	chr4:2278462	*ZFYVE28*	Body	5.14E-06	0.1	0.75	0.81	−0.05
cg04922803	chr12:104444418	*GLT8D2*	TSS1500	5.19E-06	0.1	0.68	0.62	0.06
cg18857062	chr6:43276478	*CRIP3*	Body	5.29E-06	0.1	0.21	0.26	−0.04

Listed are all probes associated with schizophrenia (SZ) with FDR ≤0.1. The 100 top-ranked DMPs are listed in Table S4 in Additional file 1.

### Region-based analysis of altered DNA methylation in schizophrenia

As DNA methylation is often correlated across adjacent CpG sites [20,21], we used the Illumina Methylation Analyser (IMA) [22] package to aggregate and assess DNA methylation across adjacent probes within annotated regions of the genome. Again, we observe widespread evidence for schizophrenia-associated DNA methylation differences, occurring specifically in the PFC (Figure S1 and Table S6 in Additional file 1). Most notably we observe a large (approximately 8 kb) region spanning the gene body of the *Neuritin 1* (*NRN1*) gene across which 29 adjacent CpG sites are consistently hypomethylated in schizophrenia patients compared with controls (Figure 1B). The mean DNA methylation difference across all 29 sites is -1.77% (FDR = 3.1e-2), with a notable region of hypomethylation (-3.7%) spanning three consecutive DMPs (FDR = 2.4e-2). This is of particular interest because *NRN1* plays a well-established role in neurodevelopment and synaptic plasticity [23], and genetic variation in the gene has been linked to cognitive phenotypes in schizophrenia [24].

### Validation and replication of schizophrenia-associated DNA methylation differences in the prefrontal cortex

To confirm the array data, bisulfite-pyrosequencing was used to validate schizophrenia-associated DMPs in the vicinity of three genes (*NRN1* (cg00565348), *C8A* (cg23844013), and *RASA3* (cg24803255)) in the same samples. Bisulfite-PCR amplification was performed in duplicate using the primers and assay conditions in Table S7 in Additional file 1. Fully methylated and fully unmethylated control samples were included in all experiments. For each amplicon we confirmed significant DNA methylation differences in the same direction as reported by the 450K array (Figure 1C; Figure S2 and Table S8 in Additional file 1). We subsequently generated a replication PFC (BA9) 450K dataset using schizophrenia and control brains archived at the Douglas Bell-Canada Brain Bank, Montreal, Canada (n = 33, 18 schizophrenia and 15 controls; Tables S9 and S10 in Additional file 1) using the Illumina 450K array as described above. Strikingly, DNA methylation differences at the top-ranked PFC DMPs identified in the ‘LBBND’ cohort (listed in Table S11 in Additional file 1) were strongly correlated with schizophrenia-associated differences at the same probes in PFC tissue from the Montreal replication samples (r = 0.54, *P* = 6.8e-09) with a consistent direction of effect observed across both cohorts and significant differences observed for top-ranked DMPs (Figure 1C,D).

### Evidence for schizophrenia-associated gene co-methylated modules in the prefrontal cortex

**Figure 2:**
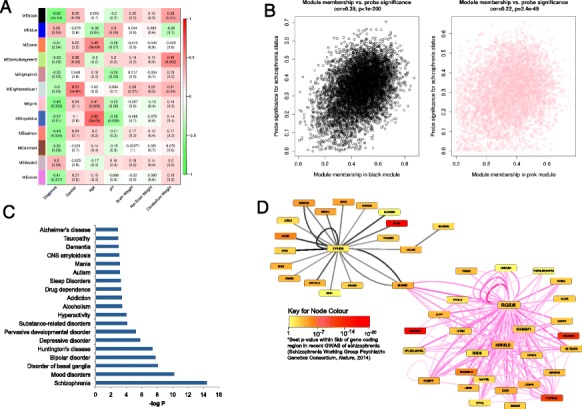
**Schizophrenia-associated gene co-methylation modules in the prefrontal cortex. (A)** Heatmap representing correlations between module eigenvalues (ME) and clinical/demographic variables. Each row represents a specific module, indicated by an arbitrary module color labeled on the y-axis. Dark red indicates strong positive correlation, dark green indicates strong negative correlation, and white indicates no correlation, as indicated in color scale bar. *P*-values are given in parentheses. The black (*P* = 0.0004) and pink (*P* = 0.002) WGCNA modules are most significantly associated with schizophrenia in the PFC. **(B)** Module membership in both modules is strongly correlated with probe significance with disease (black module: r = 0.39, *P* <1e-200; pink module: r = 0.22, *P* = 2.4e-49). **(C)** Ingenuity Pathway Analysis of genes associated with CpG sites in the black module reveals a highly significant enrichment of disease pathways related to schizophrenia and other neuropsychiatric disease. **(D)** Co-methylation networks between the top 1% of loci in the black and pink modules ranked by their module membership, where thicker lines indicate more connected genes. Black edges represent co-methylation between probes in the black module and pink edges represent co-methylation in the pink module; thicker and deeper colored edges indicate stronger correlations between probes. Interestingly, the two modules are connected by *SHANK2*, which encodes a molecular scaffold protein that plays a role in synaptogenesis. Furthermore, several of the genes are associated with schizophrenia in recent collaborative genome-wide association studies (GWAS) [27]. GWAS significance for SNPs in the vicinity of each locus is depicted by color.

We next employed weighted gene co-methylation network analysis (WGCNA) to undertake a systems-level view of the DNA methylation differences associated with schizophrenia in the PFC [25,26]. Using probe-wise DNA methylation data we identified 110 modules (representing discrete networks of co-methylated sites), and the first principal component of each individual module (termed the ‘eigengene’) was used to assess the relationship with disease status. Twelve PFC modules were significantly associated with schizophrenia, most notably the ‘black’ (n = 6,647 probes, r = -0.52, *P* = 0.0004) and ‘pink’ modules (n = 4,399 probes, r = -0.45, *P* = 0.002) (Figure 2A). Module membership in both modules is strongly correlated with probe-level disease significance (black module: r =0.39, *P* <1e-200; pink module: r = 0.22, *P* = 2.4e-49) (Figure 2B). Repeating WGCNA on the cerebellum data yielded no schizophrenia-associated modules, providing further evidence of brain region-specific DNA methylation differences in schizophrenia. Furthermore, a comparison of modules across the two brain regions showed that while a subset of 43 PFC modules correlated strongly (r >0.8) with 51 cerebellum modules, none of the PFC schizophrenia-associated modules were correlated with modules in the cerebellum. Finally, despite the low number of samples and lack of power for replicating WGCNA associations in the Montreal replication dataset, the modular structure of DNA methylation in the PFC was found to be strongly preserved across both brain cohorts (Figure S3 in Additional file 1), with both the black and pink modules being highly conserved and negatively correlated with schizophrenia (black module: r = -0.26; pink module: r = -0.10).

### Schizophrenia-associated co-methylated modules are enriched for neurodevelopmental pathways and loci previously implicated in the disorder

To test whether the identified schizophrenia-associated modules were biologically meaningful, pathway and gene ontology analyses were performed using Ingenuity Pathway Analysis (IPA) and EASE (DAVID) software [28]. Enrichment for specific pathways and biological functions was determined relative to the relevant microarray gene list using a right-tailed Fisher’s test. IPA of genes associated with CpG sites in the top-ranked PFC modules reveals a highly significant enrichment of disease pathways related to ‘schizophrenia and other neuropsychiatric disorders’ in the black module (*P* = 3.67e-15 to 1.27e-03; Figure 2C), and ‘nervous system development and function’ in both the black (*P* = 3.80e-18 to 1.37e-03; Figure S4 in Additional file 1) and pink (*P* = 4.04e-15 to 3.16e-03; Figure S5 in Additional file 1) modules. Consistent with the IPA results, gene ontology analysis using EASE (DAVID) [28] shows that both modules are significantly enriched for functions related to nervous system and neuron development, synaptic transmission and calcium ion binding (Table S12 in Additional file 1). An analysis of co-methylation networks between the top-ranked ‘hub’ probes in the black and pink modules shows that they are connected by DNA methylation in the vicinity of *SHANK2*, a molecular scaffold protein that plays a critical role in neurodevelopment and synaptogenesis, and has been widely implicated in several neurodevelopmental disorders, including schizophrenia [29] (Figure 2D). Furthermore, several of the probes are located in genes that have been strongly implicated in schizophrenia by a recent large collaborative genome-wide association study (GWAS; for example, *CACNA1C*, *TSNARE1*, *PITPNM2*, and *HLA-L*) [27].

### Schizophrenia-associated DMPs are enriched for CpG sites undergoing epigenetic changes during fetal neocortex brain development

**Figure 3:**
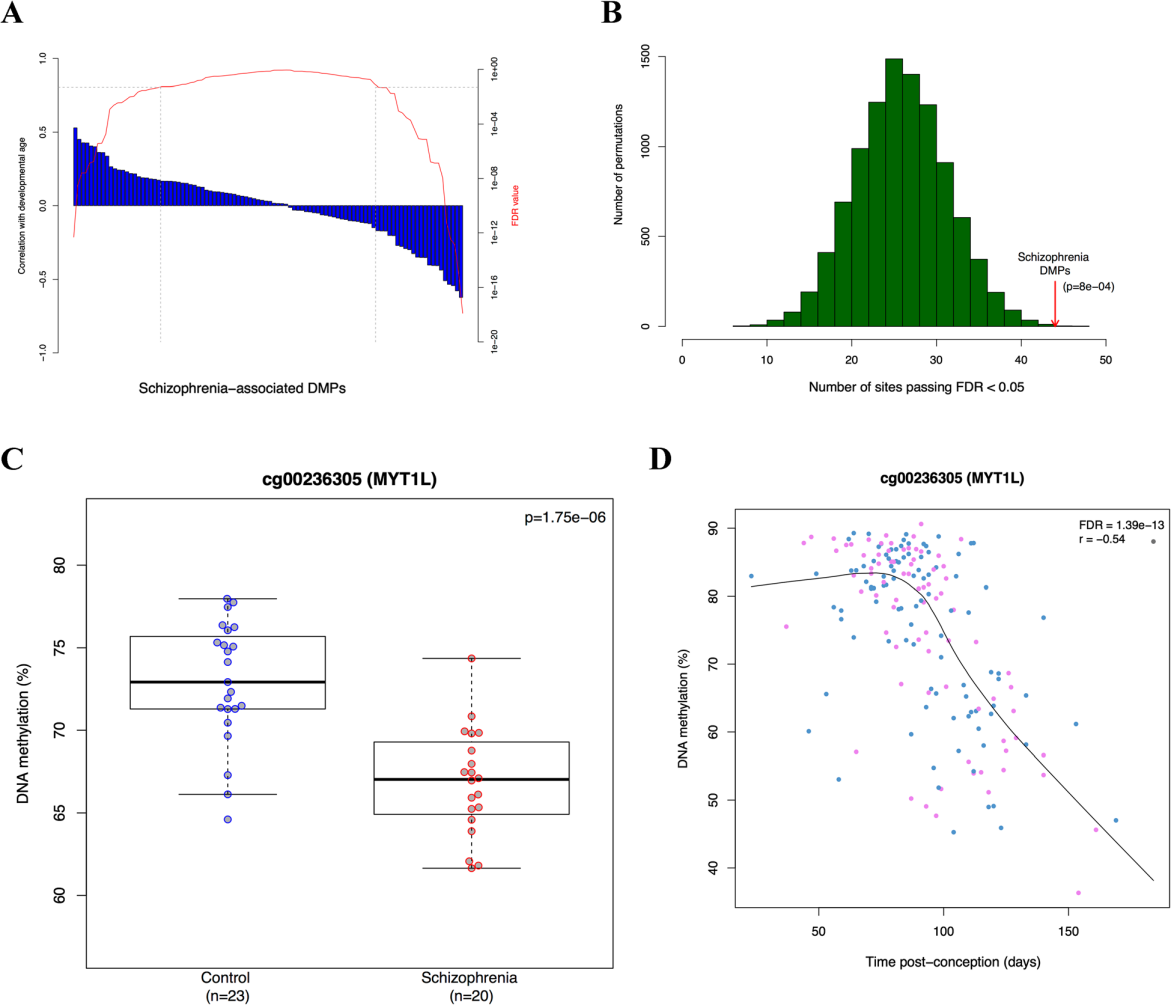
**Schizophrenia associated DMPs are enriched for CpG sites undergoing epigenetic changes during fetal neocortex brain development. (A)** Correlation between DNA methylation and neurodevelopmental age (days post-conception) for 98 of the 100 top-ranked schizophrenia-associated DMPs (blue) with corresponding FDR values (red). Dashed lines demarcate DMPs with FDR <0.01. **(B)** Distribution of the number of CpG sites significantly associated with neocortex brain development (FDR <0.05) using 10,000 permutations of 98 randomly selected CpG sites. The red arrow represents the number of significant (FDR <0.05) age-correlated CpG sites (n = 44) among the 100 top-ranked schizophrenia-associated DMPs. **(C,D)** A CpG site in *MYT1L*, for example, significantly hypomethylated in schizophrenia (*P* = 0.00000175) **(C)** is highly correlated with neocortical development (FDR = 1.39e-13) **(D)**. Blue points represent male samples and pink points represent female samples.Other examples of schizophrenia-associated DMPs correlated with neocortical development are shown in Figure S6 and Table S13 in Additional file 1.

Given previous evidence for a neurodevelopmental basis to schizophrenia [2], and our analyses demonstrating an enrichment of neurodevelopmental pathways involved in disease, we next investigated whether schizophrenia-associated DMPs are enriched for CpG sites undergoing dynamic DNA methylation changes during human fetal brain development. For each of the top-ranked PFC DMPs we assessed the correlation between DNA methylation and days post-conception in a 450K DNA methylation dataset generated by our lab using human fetal brain tissue (n = 179, range = 23 to 184 days post-conception, number of matching 450K probes in fetal dataset after quality control and filtering = 98/100; HH Spiers *et al*., in preparation). Strikingly, DNA methylation at 44% of the schizophrenia-associated DMPs is significantly associated (at FDR <0.05) with post-conception age in the developing fetal brain (Figure 3A), reflecting a highly significant enrichment for neurodevelopmental DMPs (10,000 permutations, *P* = 8e-04) amongst schizophrenia-associated loci (Figure 3B) and reinforcing the notion that neurodevelopmental dysfunction is involved in schizophrenia. For example, one of the top-ranked schizophrenia-associated DMPs (cg00236305), located in the gene body of *MYT1L*, is significantly hypomethylated (*P* = 1.75e-6) in patients compared with controls (Figure 3C). *MYT1L* encodes a potent transcription factor integral to neurodevelopment [30], and the same probe is highly correlated with neocortical development (FDR = 1.39e-13; Figure 3D). Other examples of schizophrenia-associated DMPs strongly correlated with neocortical development are shown in Figure S6 and Table S13 in Additional file 1.

## Conclusions

In this study we have found schizophrenia-associated variation in DNA methylation in the PFC, and identified discrete modules of co-methylated loci associated with the disorder that are significantly enriched for genes involved in neurodevelopmental processes. Methylomic profiling in human fetal cortex samples confirmed that disease-associated DMPs are significantly enriched for loci at which DNA methylation is dynamically altered during human fetal brain development. These data strongly support the hypothesis that schizophrenia has an important early neurodevelopmental component, and suggest that epigenetic mechanisms may mediate the relationship between neurodevelopmental disturbances and risk of disease.

## Materials and methods

### Samples and sample processing

#### London Brain Bank for Neurodegenerative Disorders

PFC and cerebellum samples were obtained from 47 brains archived in the LBBND. Subjects were approached in life for written consent for brain banking, and all tissue donations were collected and stored following legal and ethical guidelines (NHS reference number 08/MRE09/38; the HTA license number for the LBBND brain bank is 12293). The study is also approved by the University of Exeter Medical School Research Ethics Committee (reference number 13/02/009). Samples were dissected by a trained neuropathologist, snap-frozen and stored at -80°C. Schizophrenia patients were diagnosed by trained psychiatrists according to Diagnostic and Statistical Manual of Mental Disorders (DSM) criteria. Demographic information for the samples is summarized in Tables S1 and S2 in Additional file 1. Samples were randomized with respect to gender and disease status to avoid batch effects throughout all experimental procedures. Genomic DNA was isolated using a standard phenol-chloroform extraction protocol. DNA was tested for degradation and purity using spectrophotometry and gel electrophoresis.

#### Douglas Bell-Canada Brain Bank, Montreal

PFC samples from 18 schizophrenia cases and 15 controls were obtained from the Douglas Bell-Canada Brain Bank (DBCBB) [31]. Brain specimens used in this study were collected postmortem following consent obtained with next of kin, according to tissue banking practices regulated by the Quebec Health Research Fund [32], and based on the OECD Guidelines on Human Biobanks and Genetic Research Databases [33]. Samples were dissected by neuropathology technicians, snap-frozen and stored at -80°C. Psychiatric diagnoses were based on best-estimate diagnostic procedures, following SCID I diagnostic interviews conducted with informants, as described elsewhere [34]. Demographic information for the DBCBB samples is summarized in Tables S8 and S9 in Additional file 1. Genomic DNA was isolated using a standard phenol-chloroform extraction protocol. DNA was tested for degradation and purity using spectrophotometry and gel electrophoresis.

### Genome-wide quantification of DNA methylation

#### Microarray processing

DNA (500 ng) from each sample was treated with sodium bisulfite in duplicate, using the EZ-96 DNA methylation kit (Zymo Research, Irvine, CA, USA). DNA methylation was quantified using the Illumina Infinium HumanMethylation450 BeadChip (Illumina Inc.) run on an Illumina HiScan System (Illumina) using the manufacturers’ standard protocol.

#### Data processing and quality control of discovery data

Signal intensities for each probe were extracted using Illumina GenomeStudio software and imported into R [35] using the *methylumi* and *minfi* packages [36,37]. Multi-dimensional scaling plots of variable probes on the sex chromosomes were used to check that the predicted gender corresponded with the reported gender for each individual. One sample showed a discrepancy between measured and reported sex and was subsequently identified as having an XXY karyotype and removed from the current study for independent investigation [38]. Comparison of non-CpG SNP probes on the array confirmed that matched PFC and cerebellum tissues were sourced from the same individual. Raw beta values of probes within brain region-specific DMRs (extracted from [39]) were used to confirm the tissue identity of each sample. Probes containing a SNP with minor allele frequency >5% within 10 bp of the single base extension site based on Illumina’s database and probes identified by Chen and colleagues [40] (n = 34,548) were removed from all analyses. Further data quality control and processing steps were conducted using the *wateRmelon* package [16] in R. The *pfilter* function was used to filter data by beadcount and detection *P*-value. Samples with >1% probes with a detection *P*-value >0.05 were removed (PFC: n = 3 samples; cerebellum: n = 1 sample). Probes with a detection *P*-value >0.05 in at least 1% of samples and/or a beadcount <3 in 5% of samples were removed across all samples to stringently control for poor quality probes. Quality control sample exclusions are summarized in Table S3 in Additional file 1. The *dasen* function was used to normalize the data as previously described [16], with probes on the sex chromosomes removed from all subsequent analysis.

#### Statistical analysis

First, analyses were performed to test for DNA methylation differences between schizophrenia cases and controls at the individual probe level. To model the effect of sample-specific variables we performed linear regression for each probe using age, gender and disease status as independent variables. Regression tests were performed using the *Limma* [41] package in R, and prior to analyses β-values were log-transformed to M-values to improve sensitivity. *P*-values were adjusted for multiple testing according to the FDR procedure of Benjamini-Hochberg. The CETS package in R [19] was used to check that our top-ranked DMPs were not mediated by the effect of differential neuronal cell proportions across samples. To identify DMRs we used the Illumina Methylation Analyzer (IMA) [22] package to compute region-level summaries of DNA methylation in the PFC and cerebellum, which were then tested for association with disease using *Limma. P*-values were adjusted for multiple testing according to the FDR procedure of Benjamini-Hochberg. Significant DMRs were selected at a 5% FDR and visualized using *RCircos* [42] and *Gviz* [43] packages. To investigate whether schizophrenia-associated DMPs are enriched for sites undergoing epigenetic changes during neurodevelopment, we used an unpublished 450K DNA methylation dataset of fetal cortex brain samples (n = 179, range 23 to 184 days post-conception) collected as part of ongoing work in our group (HH Spiers *et al*., in preparation). For the top 100 schizophrenia-associated DMPs in our dataset, we extracted β-values of 98 matched probes in the neurodevelopmental dataset (n = 2 probes removed during quality control of fetal dataset). We then used Pearson’s correlation tests to assess the number of probes showing a significant relationship (FDR <0.05) between DNA methylation and days post-conception. To evaluate the genome-wide significance of this result we compared the above correlation with an estimated null distribution from 10,000 randomizations of the data. On each randomization we randomly selected 98 probes and computed the number of probes with FDR <0.05, as described above. One-tail significance was assessed by comparing the original number of significant probes (FDR <0.05) to the estimated null distribution.

### Bisulfite-pyrosequencing

Independent verification analysis was performed on three PFC schizophrenia-associated DMPs (in the vicinity of *NRN1*, *C8A*, and *RASA3*), based on results from both the probe-wise and region-level analysis. Pyrosequencing assays were designed using the PyroMark Assay design software (Qiagen, Hilden, Germany). Bisulfite-PCR amplification was performed in duplicate using the primers and assay conditions in Table S7 in Additional file 1. Fully methylated and fully unmethylated control samples were included in all experiments.

### Replication analysis

DNA samples from the Montreal cohort were analyzed using the Illumina Infinium HumanMethylation450 BeadChip using the same processing and quality control steps as described above. For the top 100 schizophrenia-associated DMPs in the LBBND cohort (Table S4 in Additional file 1), we extracted β-values for matched probes in the Montreal dataset. Pearson’s correlation was used to assess the relationship between disease-associated DNA methylation differences in the two datasets at these probes.

### Weighted gene co-methylation network analysis

Network analysis was performed on normalized probewise DNA methylation data (PFC: n = 445,617 probes; cerebellum: n = 440,836 probes) using WGCNA [26]. For each brain region pair-wise correlations were used to identify modules of highly co-methylated probes, independent of disease status. Specifically, an unsigned network was created with a soft threshold parameter of 6 using the *blockwiseModules* function based on a block size of 20,000. Each module was then labeled with a unique color name. The first principle component of the methylation matrix of each module was calculated to give a ‘module eigengene’ (ME) for each module (a weighted average methylation profile). To identify modules associated with schizophrenia, each of the ME values was regressed on disease status, in addition to other sample-specific variables: pH, age, gender, brain weight and cerebellum weight. To test whether the identified schizophrenia-associated modules were biologically meaningful, pathway and gene ontology analyses were performed using IPA [44] and EASE (DAVID) [28,45]. Correlations were performed between the ME and DNA methylation values to calculate the module membership of each probe (the extent to which a given probe contributes to the ME). This module membership value was used to identify the ‘hub’ genes in each module. The function *modulePreservation* in the WGCNA package was used to calculate module preservation statistics for the Montreal PFC data based on the PFC modules built from the discovery dataset.

### Accession numbers

The Gene Expression Omnibus accession numbers for the 450K array data reported in this paper are GSE61431 (LBBND dataset) and GSE61380 (DBCBB dataset).

## Additional file

Additional file 1:
**Supplementary Figures S1 to S6 and Supplementary Tables S1 to S13.**

## Abbreviations

DBCBB: Douglas Bell-Canada Brain Bank
DMP: differentially methylated position
DMR: differentially methylated region
FDR: false discovery rate
GWAS: genome-wide association study
IPA: Ingenuity Pathway Analysis
LBBND: London Brain Bank for Neurodegenerative Diseases
ME: module eigengene
PFC: prefrontal cortex
SNP: single-nucleotide polymorphism
UTR: untranslated region
WGCNA: weighted gene co-methylation network analysis
