# Supplementary material for: Methylomic profiling of human brain tissue supports a neurodevelopmental origin for schizophrenia
Source: Genome Biol. 2014 Oct 28;15(10):483. doi: 10.1186/s13059-014-0483-2 (PMC4262979; doi:10.1186/s13059-014-0483-2)
Supplement: Additional file 1: — Supplementary Figures S1 to S6 and Supplementary Tables S1 to S13. [file 13059_2014_483_MOESM1_ESM.pdf]

SUPPLEMENTARY FIGURE 1 – Circos plot of schizophrenia-associated differentially methylated regions (DMRs) in the prefrontal cortex. The outermost ring denotes chromosomal location (annotated with chromosomal number), with the pter-qter orientation in a clockwise direction. Small red lines represent the centromeres within each chromosome. The middle ring highlights the location of genes associated with schizophrenia-associated DMRs (FDR ≤ 0.05), with the magnitude and direction of DNA methylation difference for each region shown in the inner ring. Lines pointed inwards signify hypomethylated regions and outwards hypermethylated regions. Line colors indicate the genomic region or CpG island location of the probe. TSS=Transcription start site; UTR=Untranslated Region.

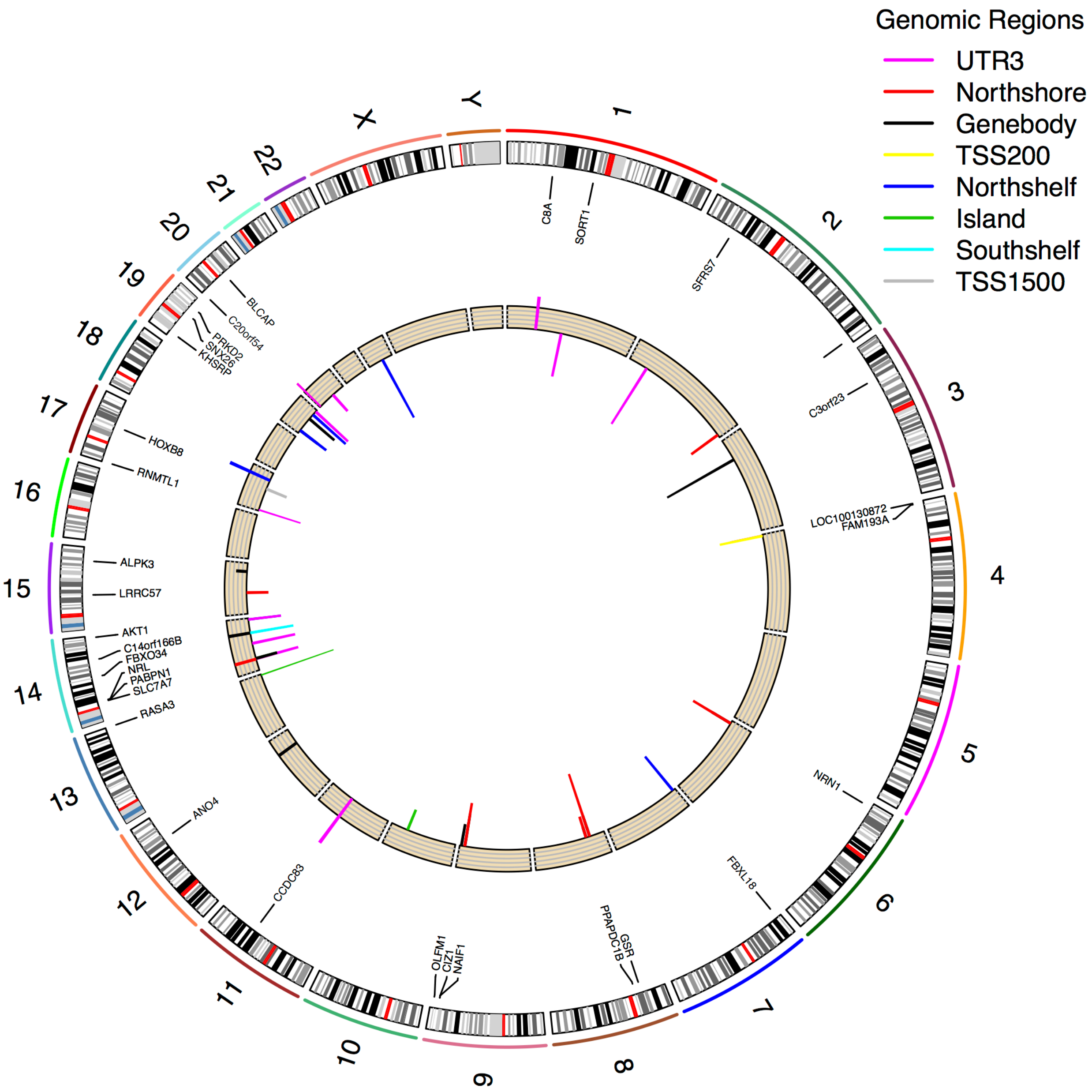

SUPPLEMENTARY FIGURE 2 - Validation of Illumina 450K array data using bisulfite-pyrosequencing. Shown is confirmation of schizophrenia-associated DNA methylation differences at a) cg23844013 *(C8A)*, b) cg00565348 *(NRN1)*, and c) cg24803255 *(RASA3)*. Additional validation data is presented in **Figure 1c**.

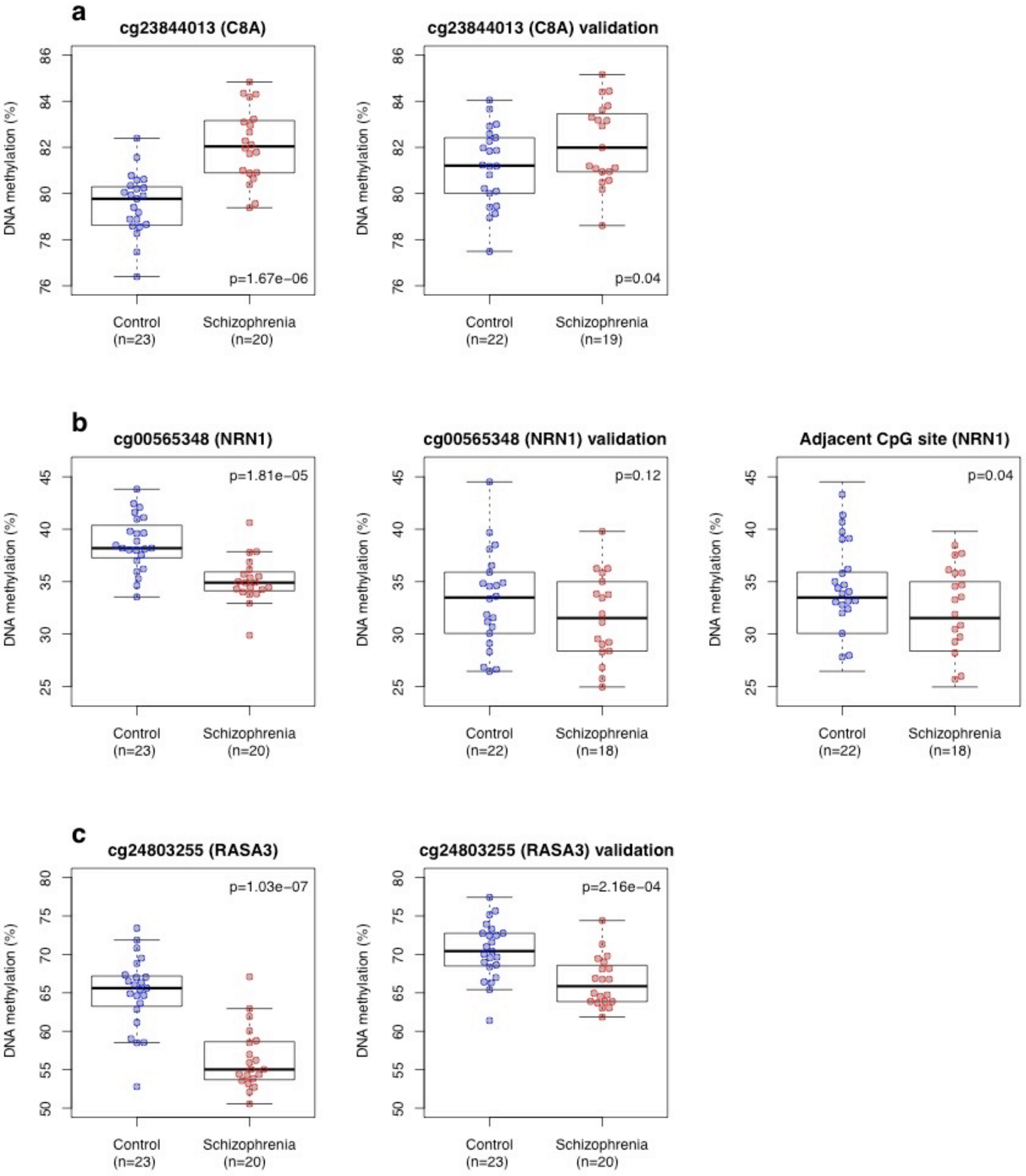

SUPPLEMENTARY FIGURE 3 – Modules of co-methylated loci identified in the 'discovery' PFC cohort by WGCNA are strongly preserved in the Montreal replication cohort. A Zsummary score > 10 is indicative of a strongly preserved module. Of note, the two modules associated with schizophrenia are highly conserved across datasets (black module: Zsummary score = 190; pink module: Zsummary score = 49).

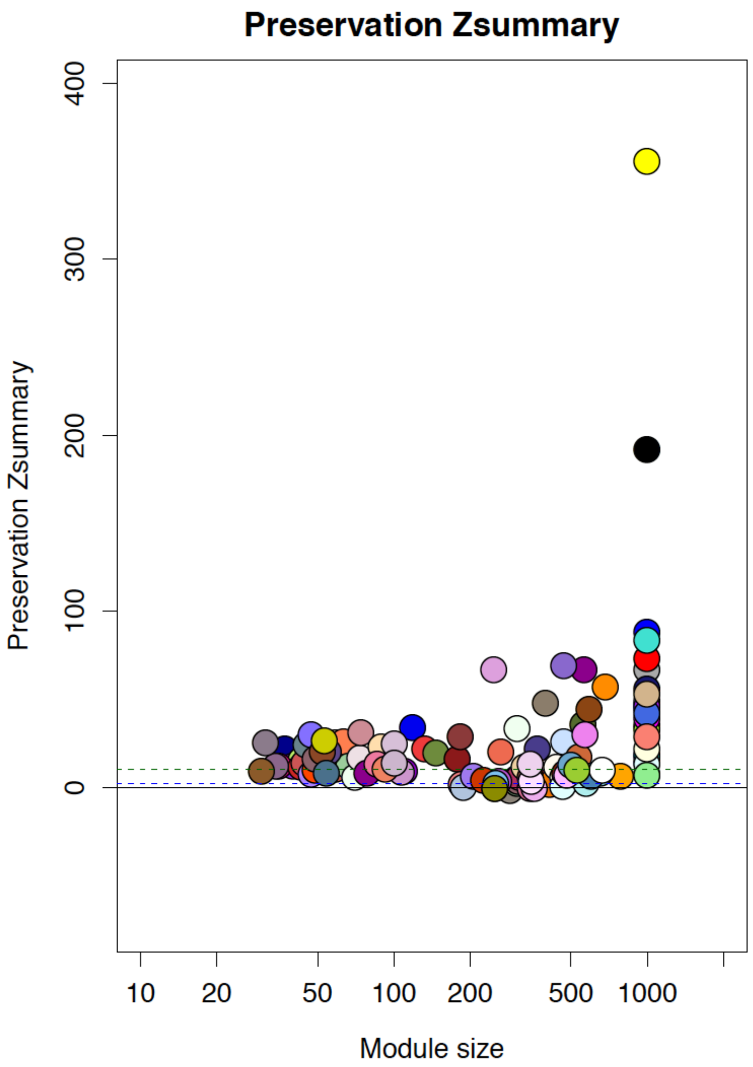

SUPPLEMENTARY FIGURE 4 - Ingenuity Pathway Analysis highlighted a highly significant (p=3.80E-18 to 1.37E-03) enrichment of pathways related to nervous system development and function in the black WGCNA module.

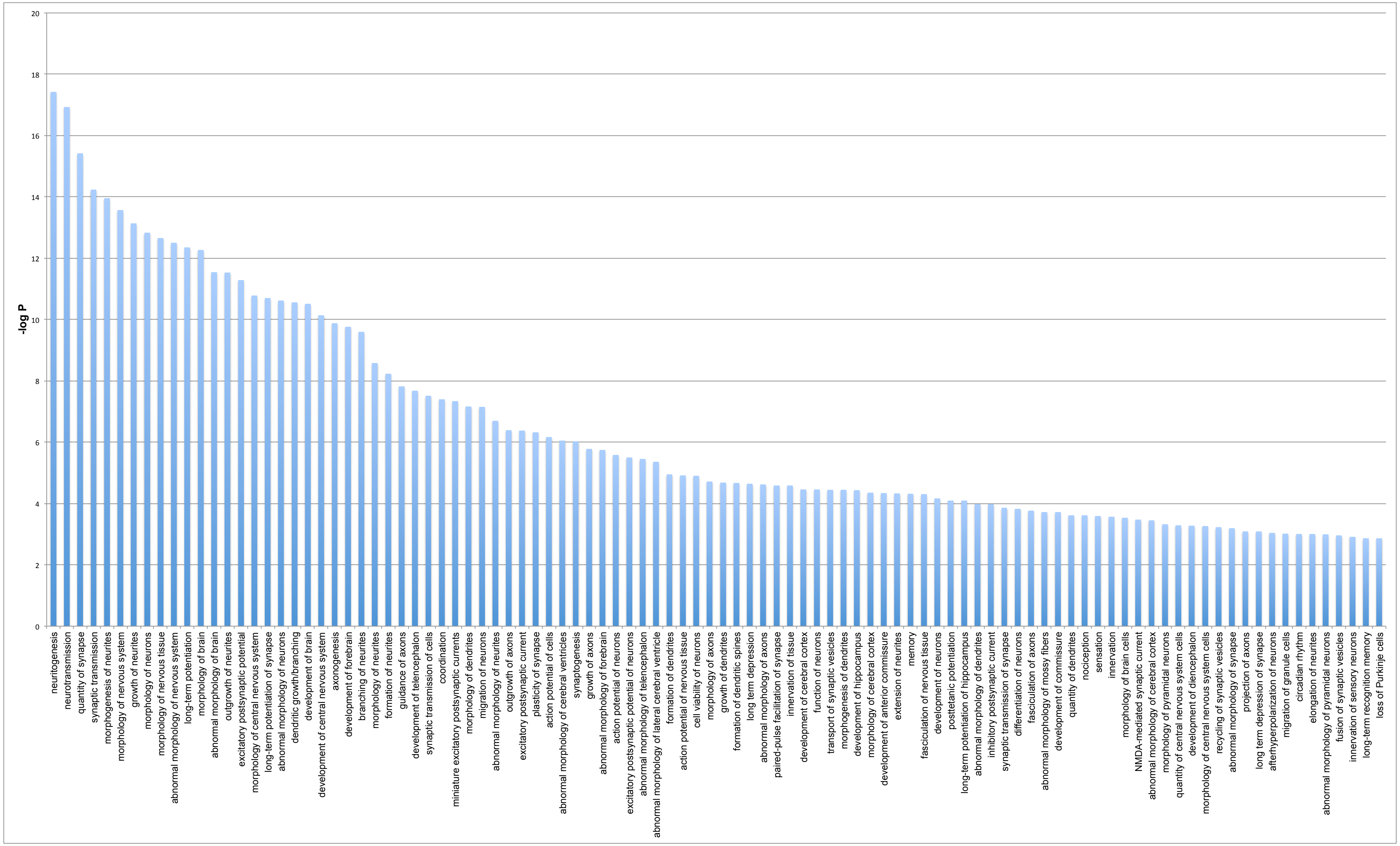

SUPPLEMENTARY FIGURE 5 - Ingenuity Pathway Analysis highlighted a highly significant (p=4.04E-15 to 3.16E-03) enrichment of pathways related to nervous system development and function in the pink WGCNA module.

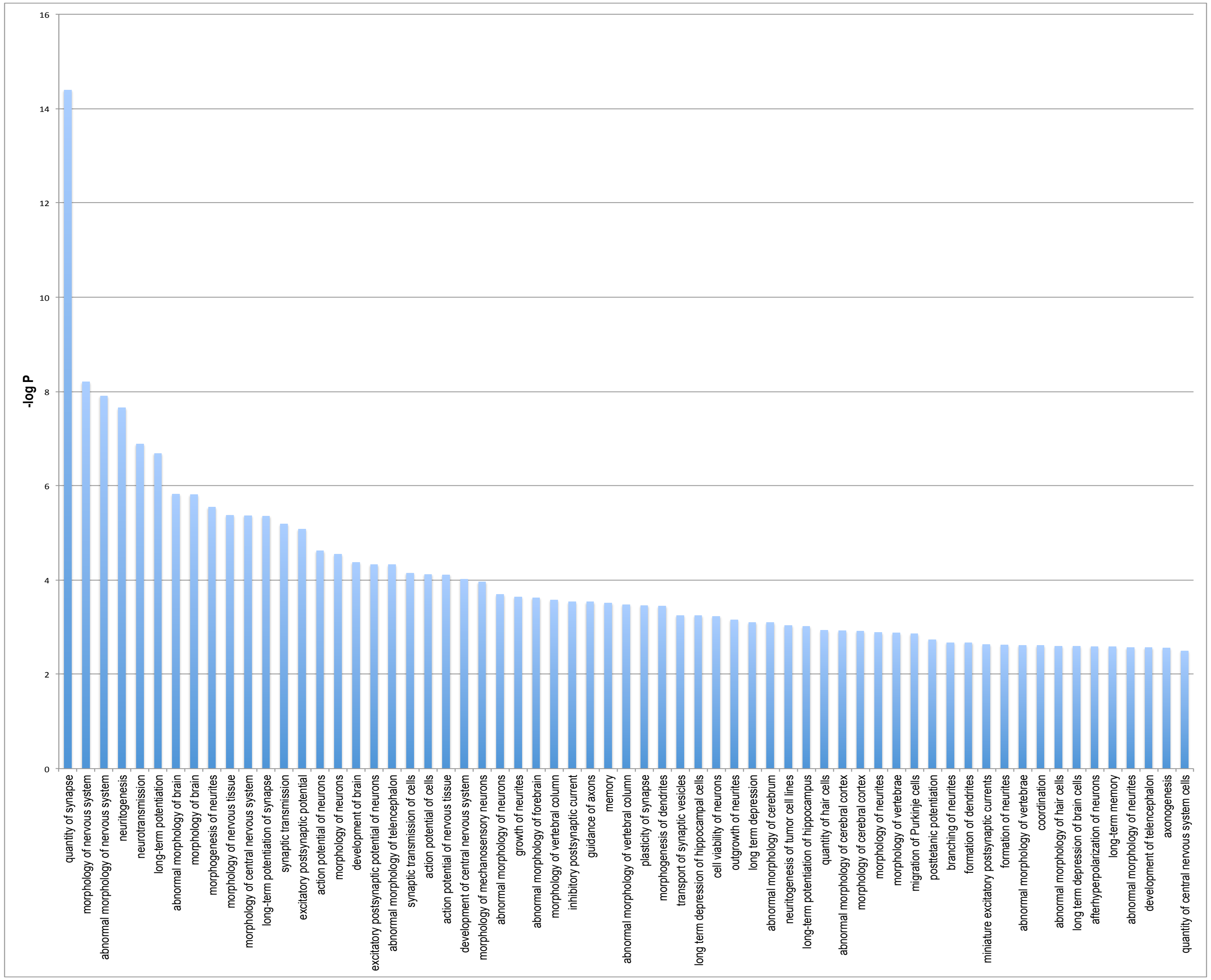

SUPPLEMENTARY FIGURE 6 - Schizophrenia-associated DMPs are enriched for loci showing dynamic changes in DNA methylation during human brain development. Correlation between DNA methylation and neurodevelopmental age (days post-conception) is shown for top-ranked schizophrenia-associated DMPs that are also associated with brain development (**Supplementary Table 13**).

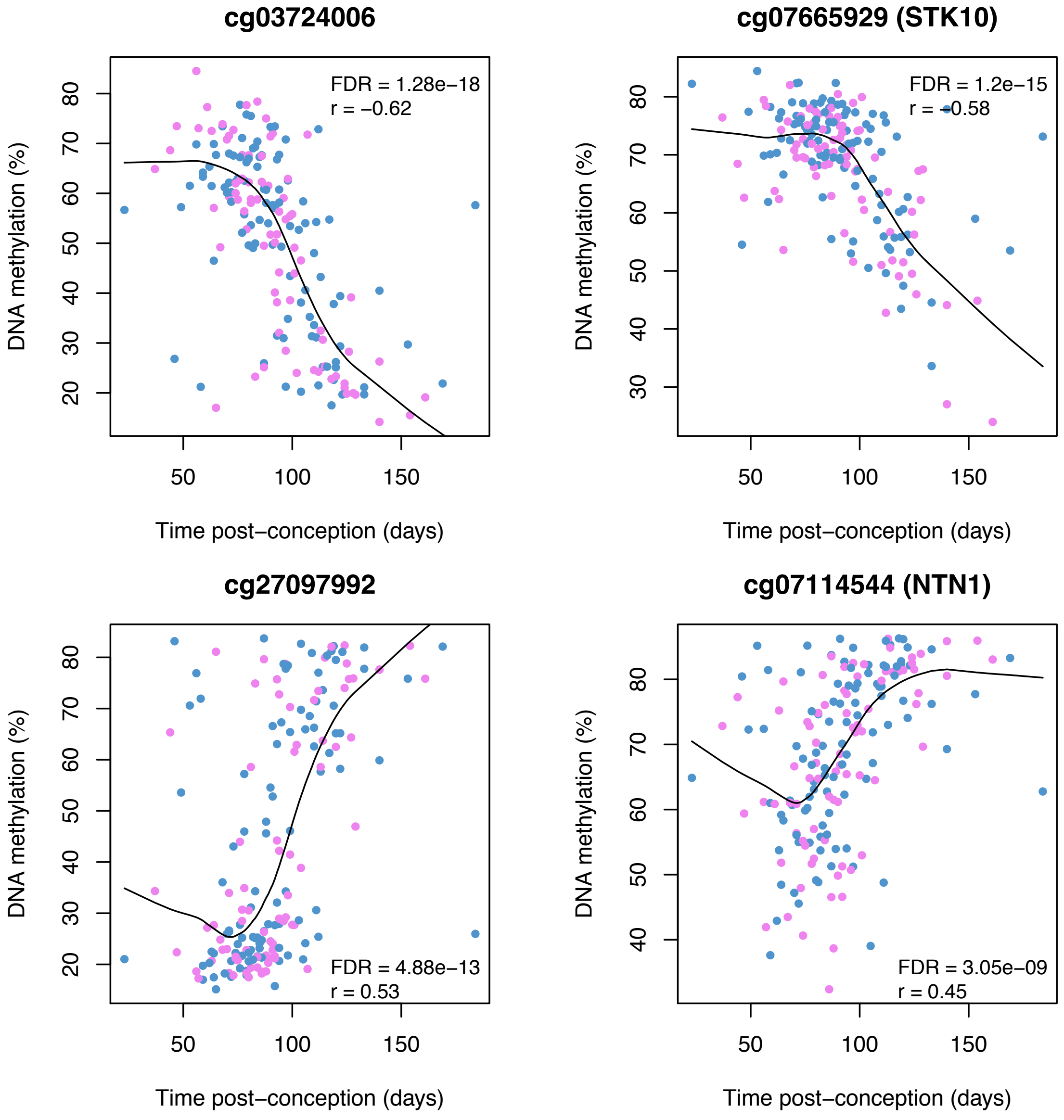

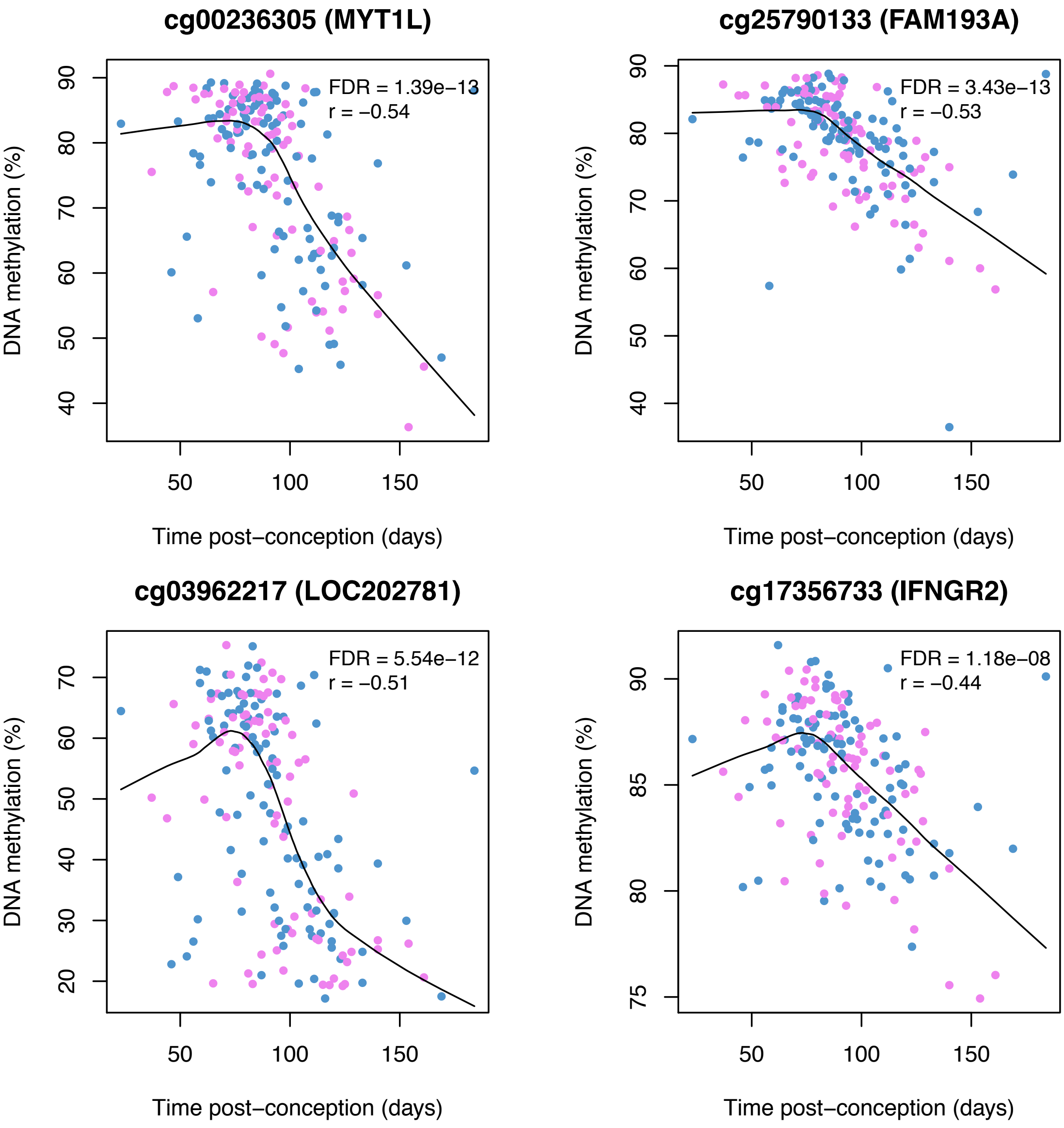
cg00236305 (MYT1L)
FDR = 1.39e−13
r = −0.54
cg25790133 (FAM193A)
FDR = 3.43e−13
r = −0.53
cg03962217 (LOC202781)
FDR = 5.54e−12
r = −0.51
cg17356733 (IFNGR2)
FDR = 1.18e−08
r = −0.44
DNA methylation (%)
Time post−conception (days)

SUPPLEMENTARY TABLE 1 - Details of post-mortem brain samples from London Brain Bank of Neurodegenerative Disease. Schizophrenia cases and controls are matched (t-test p-value>0.05) for all variables except total brain weight. SZ=schizophrenia.

	SZ	Control	Total	p-value
**Number**	22	24	46	
**Sex (male:female)**	12:10	18:6	30:16	
**Age at death (years)**	61± 16.6	61.1 ± 18.9	61.1 ± 17.6	0.98
**Total brain weight (g)**	1354± 143	1465 ± 193	1416 ± 180	0.04**
**Net brain weight (g)**	1215± 132	1292 ± 173	1260 ± 160	0.11
**Cerebellum weight (g)**	164 ± 15.5	175 ± 28	171 ± 24	0.10
**pH**	6.60 ± 0.29	6.48 ± 0.33	6.54 ± 0.32	0.19

SUPPLEMENTARY TABLE 2 - Details of individual post-mortem brain samples from the London Brain Bank of Neurodegenerative Disease. NA=not available.

ID	Sex	Age at death (years)	Total brain weight (g)	Net brain weight (g)	Cerebellum weight (g)	pH
Schizophrenia						
**ID1**	M	67	1487	1313	174	6.36
**ID2**	M	79	NA	NA	NA	6.40
**ID3**	F	69	NA	NA	NA	6.80
**ID4**	F	76	1321	1143	178	6.25
**ID5**	F	84	1201	NA	NA	6.60
**ID6**	M	87	1126	993	133	6.19
**ID7**	F	32	1356	1185	171	6.30
**ID8**	F	70	1340	1193	147	6.17
**ID9**	F	49	1535	1354	181	6.90
**ID10**	M	46	1283	1117	166	7.00
**ID11**	M	62	1614	1439	175	7.00
**ID12**	M	31	1273	1273	NA	6.30
**ID13**	M	51	1663	1485	178	6.80
**ID14**	M	62	NA	NA	NA	7.10
**ID15**	F	34	1237	1092	145	6.20
**ID16**	F	75	1314	1141	173	6.60
**ID17**	M	64	1239	NA	NA	6.70
**ID18**	M	49	1263	1097	166	6.80
**ID19**	F	71	1283	1141	142	6.83
**ID20**	F	75	1310	1162	148	6.61
**ID21**	M	64	1375	1197	178	6.60
**ID22**	M	45	1499	1332	167	6.70
Control						
**ID23**	M	25	1731	1549	182	6.93
**ID24**	M	49	1411	1231	180	6.70
**ID25**	F	82	1376	1216	160	6.33
**ID26**	F	68	1196	1052	144	6.02
**ID27**	F	33	1503	1323	180	6.36
**ID28**	M	50	1857	1650	207	6.49
**ID29**	M	57	1287	1146	141	6.62
**ID30**	M	58	1364	1145	219	6.87
**ID31**	F	68	1305	1137	168	7.10
**ID32**	M	71	1606	1356	250	6.38
**ID33**	M	95	1310	1160	150	6.14
**ID34**	M	40	1458	1286	172	6.13
**ID35**	M	54	1487	1347	140	6.96
**ID36**	F	76	1329	1175	154	6.41
**ID37**	M	67	1505	1336	169	6.06
**ID38**	F	96	1263	1115	148	6.55
**ID39**	M	37	1389	1214	175	6.46
**ID40**	M	40	1670	1470	200	6.80
**ID41**	M	70	1175	1107	136	6.44
**ID42**	M	69	1943	1739	204	6.60
**ID43**	M	48	1450	1262	188	6.20
**ID44**	M	80	1488	1300	188	6.04
**ID45**	M	79	1598	1406	192	5.95
**ID46**	M	55	1452	1288	164	6.90

SUPPLEMENTARY TABLE 3 – Samples excluded during quality control (QC) for methylation assays. Pyro=Pyrosequencing, BC=Bisulfite Conversion, PFC=Prefrontal cortex, CER=Cerebellum, NA=not available.

ID	PFC 450K Barcode	PFC 450K QC	CER 450K Barcode	CER 450K QC	PFC NRN1 Pyro	PFC C8A Pyro	PFC RASA3 Pyro
Schizophrenia							
**ID1**	6055432012_R04C02		6042316042_R03C01				
**ID2**	6055432012_R06C01		6042316047_R02C01				
**ID3**	6055432012_R01C02		6042316024_R06C02				
**ID4**	6055432012_R01C01		6042316042_R05C01		Failed QC	Failed QC	
**ID5**	6055432066_R02C01		6042316024_R03C01				
**ID6**	6055432029_R02C02		6042316031_R03C02				
**ID7**	6055432060_R03C01		6042316024_R04C01				
**ID8**	6055432012_R04C01	Failed QC	6042316024_R04C02		NA on 450K	NA on 450K	NA on 450K
**ID9**	6055432066_R01C02		6042316031_R05C02				
**ID10**	6055432060_R04C02		6042316024_R02C02				
**ID11**	6055432066_R05C02		6042316024_R02C01				
**ID12**	6055432012_R02C01	Failed QC	6042316024_R01C01		NA on 450K	NA on 450K	NA on 450K
**ID13**	6055432060_R05C02		6042316042_R05C02				
**ID14**	6055432066_R03C02		6042316031_R01C01				
**ID15**	6055432060_R03C02		6042316047_R03C02				
**ID16**	6055432029_R05C01		6042316031_R06C01				
**ID17**	6055432029_R04C01		6042316042_R04C02				
**ID18**	6055432060_R04C01		6042316031_R02C01				
**ID19**	6055432029_R03C02		6042316031_R04C02				
**ID20**	6055432012_R03C01		6042316024_R05C02				
**ID21**	6055432066_R04C02		6042316031_R02C02				
**ID22**	6055432066_R01C01		6042316024_R03C02	Not CER	Failed QC		
Control							
**ID23**	6055432012_R06C02		6042316042_R01C02				
**ID24**	6055432012_R03C02		6042316031_R03C01				
**ID25**	6055432012_R05C01		6042316047_R06C01				
**ID26**	6055432060_R05C01		6042316024_R06C01				
**ID27**	6055432029_R02C01		6042316042_R06C02				
**ID28**	6055432066_R05C01		6042316047_R05C01				
**ID29**	6055432066_R04C01		6042316042_R02C02				
**ID30**	6055432012_R05C02		6042316031_R06C02				
**ID31**	6055432029_R03C01		6042316047_R05C02				
**ID32**	6055432012_R02C02		6042316024_R05C01		Failed QC	Failed QC	
**ID33**	6055432066_R02C02		6042316047_R04C02				
**ID34**	6055432066_R03C01	Failed QC	6042316031_R01C02		NA on 450K	NA on 450K	NA on 450K
**ID35**	6055432060_R02C02		6042316047_R04C01				
**ID36**	6055432060_R06C01		6042316042_R04C01				
**ID37**	6055432060_R01C01		6042316031_R05C01				
**ID38**	6055432060_R06C02		6042316031_R04C01				
**ID39**	6055432029_R01C02		6042316042_R03C02				
**ID40**	6055432029_R04C02		6042316042_R01C01				
**ID41**	6055432029_R05C02		6042316042_R02C01				
**ID42**	6055432060_R01C02		6042316024_R01C02				
**ID43**	6055432029_R06C01		6042316042_R06C01				
**ID44**	6055432060_R02C01		6042316047_R01C01				
**ID45**	6055432029_R06C02		6042316047_R02C02				
**ID46**	6055432029_R01C01		6042316047_R03C01	Failed BC			
**Total included in analysis:**		**43**		**44**	**40**	**41**	**43**

SUPPLEMENTARY TABLE 4 – 100 top-ranked schizophrenia-associated DMPs in the prefrontal cortex. P-value from linear model including age and sex as covariates. TSS=Transcription start site; UTR=Untranslated region, SZ=schizophrenia. The CETS algorithm[1] was used to correct for the potential effect of differential neuronal cell proportions across samples.

Probe ID	Genomic position (hg19)	Gene	Gene region	Probe type	*P*	FDR	CETS-adjusted *P*	Mean SZ	Mean Control	Beta difference
**cg26173173**	chr8:144642813*	GSDMD	Body	II	1.16E-07	0.03	2.76E-06	0.83	0.79	0.04
**cg24803255**	chr13:114807060	RASA3	Body	II	1.25E-07	0.03	1.09E-05	0.56	0.65	-0.09
**cg00903099**	chr7:154862441	HTR5A	TSS200	II	2.40E-07	0.03	1.95E-05	0.09	0.12	-0.02
**cg08171022**	chr11:70185278	PPFIA1	Body	II	2.85E-07	0.03	2.93E-05	0.48	0.53	-0.05
**cg02857643**	chr17:48696108	CACNA1G	Body	I	1.63E-06	0.1	5.88E-05	0.87	0.92	-0.05
**cg00236305**	chr2:1796076	MYT1L	Body	II	1.75E-06	0.1	1.95E-04	0.67	0.73	-0.06
**cg14966346**	chr14:104152124	KLC1	Body	II	2.51E-06	0.1	1.25E-04	0.72	0.77	-0.05
**cg13079528**	chr7:4027346	SDK1	Body	I	2.75E-06	0.1	3.07E-04	0.71	0.79	-0.08
**cg14429765**	chr8:6492531	MCPH1	Body	II	2.79E-06	0.1	2.94E-06	0.79	0.76	0.03
**cg08602214**	chr8:22864392	RHOBTB2	Body	II	2.87E-06	0.1	1.76E-04	0.69	0.75	-0.07
**cg19735533**	chr2:241196887	-	-	II	2.94E-06	0.1	2.22E-04	0.76	0.81	-0.06
**cg09507608**	chr22:29873338	-	-	II	2.95E-06	0.1	2.88E-04	0.56	0.62	-0.06
**cg23844013**	chr1:57383752	C8A	3'UTR	II	3.20E-06	0.1	4.07E-06	0.82	0.79	0.03
**cg26578910**	chr19:47204096	PRKD2	Body	I	3.47E-06	0.1	3.43E-04	0.67	0.71	-0.04
**cg21847368**	chr13:113744010	MCF2L	Body	II	3.70E-06	0.1	3.08E-05	0.80	0.84	-0.03
**cg03607729**	chr16:25138698	LCMT1	Body	II	4.19E-06	0.1	4.38E-04	0.70	0.75	-0.04
**cg10248981**	chr10:126157948	LHPP	Body	I	4.32E-06	0.1	4.81E-04	0.55	0.63	-0.07
**cg03445663**	chr2:242170236	HDLBP	Body	I	4.59E-06	0.1	4.11E-04	0.51	0.59	-0.08
**cg15079231**	chr3:42572745	VIPR1	Body	II	4.93E-06	0.1	4.18E-04	0.76	0.80	-0.04
**cg21341878**	chr4:2278462	ZFYVE28	Body	II	5.14E-06	0.1	2.71E-04	0.75	0.81	-0.05
**cg04922803**	chr12:104444418	GLT8D2	TSS1500	II	5.19E-06	0.1	1.36E-04	0.68	0.62	0.06
**cg18857062**	chr6:43276478	CRIP3	Body	I	5.29E-06	0.1	1.43E-04	0.21	0.26	-0.04
**cg27138871**	chr11:74950396	-	-	II	5.63E-06	0.11	6.10E-04	0.75	0.79	-0.05
**cg27363226**	chr19:6421747	KHSRP	Body	II	6.63E-06	0.12	7.06E-04	0.62	0.67	-0.05
**cg02187259**	chr2:16804452	FAM49A	5'UTR	II	6.72E-06	0.12	7.22E-04	0.74	0.79	-0.05

**cg26182253**	chr13:112236782	-	-	II	7.01E-06	0.12	7.68E-04	0.60	0.67	-0.06
**cg14370653**	chr9:137828210	-	-	II	7.18E-06	0.12	8.07E-04	0.75	0.80	-0.05
**cg14527262**	chr4:1202653	LOC100130 872-SPON2	TSS200	I	8.02E-06	0.12	8.77E-04	0.56	0.63	-0.07
**cg16393877**	chr17:65037838	-	-	II	8.07E-06	0.12	2.52E-04	0.78	0.74	0.04
**cg26130533**	chr4:1202700	LOC100130 872-SPON2	TSS200	II	8.92E-06	0.12	6.42E-04	0.57	0.63	-0.06
**cg05860371**	chr4:152931291	-	-	II	9.10E-06	0.12	1.02E-03	0.63	0.69	-0.06
**cg03363012**	chr20:741049	C20orf54	3'UTR	II	9.22E-06	0.12	4.94E-04	0.84	0.81	0.03
**cg21609813**	chr8:22004881	LGI3	3'UTR	I	9.89E-06	0.12	6.72E-04	0.77	0.73	0.04
**cg27608031**	chr16:77353824	ADAMTS18	Body	II	1.00E-05	0.12	1.46E-04	0.73	0.68	0.04
**cg21390682**	chr13:113743751	MCF2L	Body	II	1.01E-05	0.12	1.13E-03	0.68	0.73	-0.05
**cg27654476**	chr14:77503345	-	-	II	1.03E-05	0.12	3.98E-04	0.64	0.68	-0.04
**cg20930320**	chr1:109852598	SORT1	3'UTR	II	1.04E-05	0.12	3.86E-05	0.59	0.64	-0.04
**cg12864185**	chr1:240160972	-	-	II	1.07E-05	0.12	4.22E-05	0.17	0.22	-0.05
**cg13119928**	chr12:53228661	KRT79	TSS1500	II	1.13E-05	0.12	4.67E-04	0.81	0.78	0.03
**cg20681783**	chr5:8839868	-	-	I	1.14E-05	0.12	1.04E-03	0.38	0.33	0.05
**cg07114544**	chr17:9110369	NTN1	Body	II	1.15E-05	0.12	4.55E-04	0.73	0.69	0.04
**cg09419102**	chr11:65550444	-	-	II	1.15E-05	0.12	4.70E-04	0.70	0.66	0.04
**cg25778661**	chr19:56031373	-	-	II	1.20E-05	0.12	1.04E-03	0.79	0.84	-0.05
**cg13280041**	chr6:138866865	NHSL1	Body	I	1.22E-05	0.12	1.38E-03	0.31	0.26	0.05
**cg14772639**	chr2:128711564	SAP130	Body	II	1.28E-05	0.12	1.22E-03	0.79	0.74	0.04
**cg09942424**	chr17:74913419	MGAT5B	Body	II	1.35E-05	0.12	1.46E-03	0.78	0.82	-0.04
**cg04691233**	chr11:133989435	JAM3	Body	II	1.37E-05	0.12	1.46E-03	0.84	0.80	0.04
**cg07500432**	chr18:77918588	PARD6G	Body	II	1.37E-05	0.12	1.15E-05	0.81	0.71	0.11
**cg03991188**	chr7:98870835	MYH16	TSS200	II	1.41E-05	0.12	6.29E-05	0.82	0.78	0.04
**cg21843517**	chr12:123950174	SNRNP35	Body	II	1.41E-05	0.12	1.23E-04	0.83	0.79	0.04
**cg12198176**	chr22:23415316	RTDR1	Body	II	1.46E-05	0.12	1.58E-03	0.63	0.68	-0.05
**cg19643441**	chr19:19417142	SF4	Body	II	1.53E-05	0.12	2.85E-04	0.80	0.83	-0.03
**cg07289133**	chr16:85147146	FAM92B	TSS1500	II	1.54E-05	0.12	1.30E-03	0.74	0.69	0.05
**cg11945824**	chr6:32116963	PRRT1	3'UTR	I	1.54E-05	0.12	1.24E-03	0.58	0.64	-0.06

**cg20011075**	chr1:4008090	-	-	I	1.69E-05	0.13	1.92E-03	0.62	0.69	-0.07
**cg14752965**	chr17:17579346	-	-	II	1.72E-05	0.13	1.88E-03	0.75	0.80	-0.05
**cg07421682**	chr15:102030850	PCSK6	TSS1500	II	1.81E-05	0.13	1.94E-03	0.76	0.82	-0.05
**cg22697364**	chr5:32709859	-	-	II	1.85E-05	0.13	1.67E-03	0.26	0.22	0.04
**cg25790133**	chr4:2627014	FAM193A	TSS200	II	1.89E-05	0.13	2.06E-03	0.51	0.57	-0.06
**cg10622536**	chr1:36771021	C1orf113	TSS1500	II	1.90E-05	0.13	1.81E-03	0.77	0.81	-0.04
**cg01936649**	chr6:164062746	-	-	II	1.92E-05	0.13	1.85E-03	0.76	0.82	-0.06
**cg07665929**	chr5:171523225	STK10	Body	II	1.97E-05	0.13	2.23E-03	0.59	0.66	-0.07
**cg21375204**	chr7:73752935	CLIP2	Body	I	2.02E-05	0.13	2.19E-03	0.75	0.80	-0.05
**cg23203234**	chr8:103751623	-	-	II	2.02E-05	0.13	2.01E-03	0.71	0.76	-0.05
**cg04215055**	chr17:74911520	MGAT5B	Body	I	2.03E-05	0.13	2.12E-03	0.65	0.71	-0.06
**cg06168875**	chr19:13988438	NANOS3	1stExon	II	2.06E-05	0.13	4.21E-04	0.76	0.82	-0.06
**cg24079702**	chr2:106015771	FHL2	TSS200	I	2.08E-05	0.13	1.80E-03	0.17	0.21	-0.04
**cg25900150**	chr8:144601851	ZC3H3	Body	I	2.11E-05	0.13	1.84E-04	0.81	0.89	-0.07
**cg27097992**	chr14:104800950	-	-	I	2.15E-05	0.14	2.44E-03	0.66	0.54	0.12
**cg22060153**	chr2:101889653	SNORD89	TSS200	II	2.22E-05	0.14	4.21E-04	0.68	0.72	-0.04
**cg23996071**	chr1:60280809	HOOK1	1stExon	II	2.25E-05	0.14	4.48E-04	0.18	0.22	-0.04
**cg07815131**	chr6:99289733	-	-	II	2.27E-05	0.14	2.17E-04	0.39	0.44	-0.06
**cg05583087**	chr3:12894947	-	-	II	2.30E-05	0.14	2.65E-03	0.46	0.51	-0.06
**cg03724006**	chr11:62790783	-	-	II	2.36E-05	0.14	2.66E-03	0.48	0.54	-0.06
**cg10535500**	chr6:33398610	SYNGAP1	Body	II	2.39E-05	0.14	4.76E-04	0.43	0.47	-0.04
**cg09235936**	chr2:242170211	HDLBP	Body	I	2.41E-05	0.14	2.73E-03	0.68	0.74	-0.06
**cg12473775**	chr11:66824650	RHOD	Body	II	2.48E-05	0.14	3.09E-04	0.72	0.68	0.04
**cg27252696**	chr6:100912940	SIM1	TSS1500	I	2.51E-05	0.14	8.59E-05	0.07	0.09	-0.01
**cg00172872**	chr12:89461426	-	-	II	2.60E-05	0.14	2.42E-03	0.77	0.82	-0.04
**cg07780199**	chr19:18830892	CRTC1	Body	II	2.69E-05	0.14	3.06E-03	0.73	0.77	-0.05
**cg19671331**	chr8:143509438	-	-	I	2.70E-05	0.14	2.13E-03	0.72	0.80	-0.08
**cg26925590**	chr16:139752	C16orf35	Body	I	2.73E-05	0.14	1.43E-03	0.85	0.90	-0.05
**cg02064366**	chr17:79045526	BAIAP2	Body	I	2.79E-05	0.14	3.20E-03	0.62	0.67	-0.05
**cg17244485**	chr2:103443515	-	-	II	2.83E-05	0.14	1.58E-03	0.38	0.32	0.05

**cg23236908**	chr14:55819305	FBXO34	3'UTR	II	2.89E-05	0.14	3.26E-03	0.77	0.81	-0.04
**cg03962217**	chr7:154796431	LOC202781	Body	II	2.90E-05	0.14	1.64E-03	0.65	0.69	-0.05
**cg17356733**	chr21:34774627	IFNGR2	TSS1500	II	2.93E-05	0.14	3.33E-03	0.64	0.70	-0.06
**cg16040341**	chr15:83544284	HOMER2	Body	II	2.93E-05	0.14	2.13E-03	0.69	0.75	-0.05
**cg24038762**	chr1:87817476	-	-	II	2.95E-05	0.14	3.40E-03	0.73	0.77	-0.04
**cg05529091**	chr12:111475875	CUX2	Body	II	2.99E-05	0.14	3.25E-03	0.65	0.70	-0.05
**cg05374733**	chr12:42853791	PRICKLE1	Body	II	3.00E-05	0.14	7.73E-06	0.80	0.83	-0.03
**cg06746318**	chr7:101755186	CUX1	Body	I	3.04E-05	0.14	3.45E-03	0.70	0.76	-0.06
**cg08404546**	chr22:37910993	CARD10	Body	II	3.07E-05	0.14	5.98E-04	0.76	0.82	-0.05
**cg14905600**	chr10:134226361	PWWP2B	3'UTR	I	3.07E-05	0.14	1.13E-03	0.75	0.81	-0.06
**cg10976478**	chr2:241263838	-	-	II	3.20E-05	0.14	3.59E-06	0.84	0.80	0.04
**cg21399807**	chr2:54955581	EML6	Body	II	3.22E-05	0.14	2.58E-03	0.74	0.78	-0.04
**cg21776682**	chr8:1387801	-	-	II	3.22E-05	0.14	3.73E-03	0.68	0.73	-0.05
**cg26593722**	chr16:87376314	FBXO31	Body	I	3.28E-05	0.14	1.37E-03	0.80	0.85	-0.05
**cg04543233**	chr20:21689105	PAX1	Body	II	3.30E-05	0.14	6.38E-04	0.16	0.19	-0.04
**cg06614697**	chr1:3696285	-	-	II	3.32E-05	0.14	3.90E-04	0.62	0.67	-0.05

[1]Guintivano, J., Aryee, M. J. & Kaminsky, Z. A. *Epigenetics* 8, 290-302,(2013).

SUPPLEMENTARY TABLE 5 – 100 top-ranked schizophrenia-associated DMPs in the cerebellum. The CETS algorithm[1] was used to correct for the potential effect of differential neuronal cell proportions across samples.
TSS=Transcription start site; UTR=Untranslated region, SZ=schizophrenia.

Probe ID	Genomic position (hg19)	Gene	Gene region	Probe type	*P*	FDR	CETS-adjusted *P*	Mean SZ	Mean Control	Beta difference
**cg05695876**	chr1:201665422	NAV1	Body	II	1.87E-07	0.08	2.88E-07	0.12	0.17	-0.04
**cg05388069**	chr16:3284378	ZNF200	5'UTR	II	6.26E-06	0.97	9.91E-06	0.65	0.59	0.07
**cg07208077**	chr12:11081755	PRH2	TSS200	II	1.06E-05	0.97	2.18E-05	0.84	0.79	0.04
**cg13941830**	chr1:50891341	-	-	II	1.18E-05	0.97	1.81E-05	0.29	0.22	0.06
**cg00560072**	chr1:61750543	NFIA	Body	II	1.35E-05	0.97	3.72E-05	0.25	0.29	-0.04
**cg13299148**	chr1:32170433	COL16A1	TSS1500	II	1.72E-05	0.97	4.19E-05	0.84	0.77	0.07
**cg05922563**	chr17:40611593	ATP6V0A1	5'UTR	II	1.91E-05	0.97	5.83E-05	0.10	0.12	-0.02
**cg01655427**	chr4:109091059	LEF1	TSS1500	II	2.02E-05	0.97	2.35E-05	0.27	0.32	-0.05
**cg07236939**	chr6:110722253	DDO	Body	II	2.12E-05	0.97	5.19E-05	0.89	0.86	0.03
**cg16487621**	chr6:29395657	OR11A1	TSS200	II	2.26E-05	0.97	4.44E-05	0.89	0.86	0.04
**cg15686782**	chr7:2143886	MAD1L1	Body	II	2.65E-05	0.99	7.89E-05	0.83	0.78	0.05
**cg00499539**	chr2:113960266	PSD4	3'UTR	II	2.83E-05	0.99	8.32E-05	0.67	0.61	0.06
**cg20863949**	chr2:71213618	TEX261	3'UTR	II	3.10E-05	0.99	3.29E-05	0.82	0.75	0.06
**cg19895047**	chr8:19460197	CSGALNACT1	TSS200	II	3.37E-05	0.99	9.44E-05	0.24	0.28	-0.04
**cg22240880**	chr17:7466821	SENP3	Body	I	3.47E-05	0.99	9.64E-05	0.80	0.76	0.04
**cg14213430**	chr7:157190333	DNAJB6	Body	II	4.12E-05	1	1.06E-04	0.17	0.23	-0.06
**cg18163452**	chr1:63466707	-	-	II	4.24E-05	1	7.50E-05	0.69	0.76	-0.07
**cg05187193**	chr6:168397037	-	-	II	4.32E-05	1	9.33E-05	0.79	0.70	0.09
**cg16239296**	chr15:83736925	BTBD1	TSS1500	II	5.46E-05	1	3.31E-05	0.88	0.85	0.03
**cg22849543**	chr20:46997755	LOC284749	Body	II	5.46E-05	1	1.46E-04	0.12	0.15	-0.02
**cg03506193**	chr8:104033864	ATP6V1C1	5'UTR	II	5.52E-05	1	1.06E-04	0.31	0.38	-0.08
**cg25836061**	chr22:19939028	COMT	5'UTR	I	5.65E-05	1	1.51E-04	0.13	0.19	-0.06
**cg19421752**	chr5:1225224	SLC6A19	3'UTR	II	5.83E-05	1	1.42E-04	0.76	0.81	-0.05
**cg02398342**	chr17:80708632	TBCD	TSS1500	II	6.25E-05	1	4.25E-05	0.72	0.59	0.13
**cg14044120**	chr9:139949577	ENTPD2	TSS1500	II	6.56E-05	1	8.69E-05	0.82	0.79	0.03

**cg02294302**	chr1:47906276	FOXD2	3'UTR	II	7.23E-05	1	1.13E-04	0.76	0.71	0.05
**cg23466059**	chr18:56422682	-	-	II	7.27E-05	1	1.78E-04	0.11	0.15	-0.04
**cg21196747**	chr6:27521385	-	-	II	7.42E-05	1	2.05E-04	0.16	0.21	-0.05
**cg12728623**	chr22:19938992	COMT	5'UTR	II	7.79E-05	1	1.30E-04	0.13	0.19	-0.06
**cg06855567**	chr4:177117009	SPATA4	TSS200	II	8.29E-05	1	1.51E-04	0.12	0.15	-0.03
**cg03812681**	chr6:1397366	-	-	II	8.37E-05	1	1.85E-04	0.20	0.23	-0.03
**cg03916014**	chr16:1722352	CRAMP1L	Body	II	8.54E-05	1	9.53E-05	0.87	0.82	0.05
**cg17381305**	chr6:133226489	-	-	II	8.64E-05	1	1.29E-04	0.78	0.73	0.05
**cg15384717**	chr1:150294084	PRPF3	5'UTR	II	9.22E-05	1	1.31E-04	0.17	0.19	-0.02
**cg16671988**	chr7:148963796	ZNF783	Body	II	9.76E-05	1	8.23E-05	0.82	0.78	0.04
**cg17468663**	chr1:19048930	PAX7	Body	II	9.81E-05	1	1.43E-04	0.86	0.83	0.03
**cg02048613**	chr2:161343862	RBMS1	Body	II	1.01E-04	1	1.81E-04	0.73	0.78	-0.06
**cg13137809**	chr7:2768988	GNA12	3'UTR	I	1.02E-04	1	2.97E-04	0.82	0.78	0.04
**cg09890930**	chr12:56149396	SARNP	Body	II	1.03E-04	1	2.67E-04	0.68	0.74	-0.06
**cg02283487**	chr14:20904020	KLHL33	TSS1500	II	1.05E-04	1	3.03E-04	0.67	0.61	0.06
**cg18951674**	chr11:93641499	-	-	I	1.13E-04	1	2.63E-04	0.92	0.94	-0.02
**cg01758993**	chr20:44993436	SLC35C2	TSS1500	II	1.14E-04	1	7.03E-05	0.19	0.23	-0.04
**cg18308233**	chr11:120470179	-	-	II	1.19E-04	1	2.53E-04	0.87	0.83	0.04
**cg03723001**	chr12:117147681	-	-	II	1.21E-04	1	3.52E-04	0.22	0.28	-0.06
**cg04162223**	chr13:114164207	TMCO3	Body	II	1.23E-04	1	3.62E-04	0.68	0.65	0.03
**cg11947857**	chr19:1411919	DAZAP1	Body	II	1.24E-04	1	2.50E-04	0.09	0.11	-0.02
**cg06841648**	chr16:85479053	-	-	II	1.26E-04	1	2.15E-04	0.15	0.19	-0.04
**cg23667707**	chr7:152569949	-	-	I	1.27E-04	1	1.30E-04	0.87	0.90	-0.03
**cg25240172**	chr2:62435157	B3GNT2	5'UTR	II	1.28E-04	1	2.47E-04	0.84	0.83	0.02
**cg26894575**	chr1:153518054	S100A4	5'UTR	II	1.29E-04	1	5.40E-05	0.71	0.67	0.04
**cg07438586**	chr6:30509366	GNL1	3'UTR	II	1.29E-04	1	3.60E-04	0.15	0.19	-0.04
**cg06637938**	chr14:75390232	RPS6KL1	TSS1500	I	1.31E-04	1	5.27E-05	0.18	0.22	-0.04
**cg26108046**	chr3:183535040	MAP6D1	3'UTR	II	1.32E-04	1	2.04E-04	0.15	0.20	-0.05
**cg02765496**	chr9:71393662	FAM122A	TSS1500	II	1.42E-04	1	4.08E-04	0.68	0.64	0.04

**cg23729107**	chr17:6679264	FBXO39	TSS1500	I	1.44E-04	1	3.62E-04	0.06	0.05	0.01
**cg17009978**	chr3:183959000	VWA5B2	Body	II	1.48E-04	1	4.25E-04	0.75	0.71	0.04
**cg19248564**	chr22:43042879	CYB5R3	5'UTR	II	1.50E-04	1	2.52E-04	0.13	0.16	-0.03
**cg16046505**	chr1:246582116	SMYD3	Body	II	1.51E-04	1	4.11E-04	0.20	0.23	-0.04
**cg20751795**	chr19:58281019	ZNF586	TSS200	I	1.64E-04	1	2.59E-04	0.06	0.06	0.01
**cg11481687**	chr14:105499998	-	-	I	1.68E-04	1	4.46E-04	0.26	0.31	-0.05
**cg00102266**	chr1:121429834	-	-	II	1.68E-04	1	2.23E-04	0.85	0.82	0.03
**cg07062338**	chr10:123301036	FGFR2	Body	II	1.79E-04	1	7.14E-05	0.78	0.74	0.04
**cg00390784**	chr6:2682491	MYLK4	Body	II	1.81E-04	1	2.39E-04	0.86	0.89	-0.03
**cg13600257**	chr6:170449417	-	-	II	1.93E-04	1	1.83E-04	0.16	0.19	-0.03
**cg18003751**	chr8:1350552	-	-	II	1.93E-04	1	5.09E-04	0.81	0.84	-0.03
**cg11571585**	chr13:28365638	GSX1	TSS1500	II	1.95E-04	1	9.09E-05	0.08	0.10	-0.02
**cg10380652**	chr1:65612850	AK3L1	TSS1500	II	1.96E-04	1	2.66E-04	0.13	0.17	-0.04
**cg15613100**	chr5:72804620	-	-	II	1.96E-04	1	5.24E-04	0.78	0.73	0.05
**cg26125384**	chr2:70314274	PCBP1	TSS1500	I	1.96E-04	1	4.49E-04	0.14	0.17	-0.02
**cg27650870**	chr8:41583136	ANK1	Body	II	2.00E-04	1	3.44E-04	0.68	0.63	0.05
**cg15827217**	chr1:21833476	-	-	II	2.00E-04	1	3.58E-04	0.88	0.85	0.03
**cg00688979**	chr6:31113017	CCHCR1	Body	II	2.02E-04	1	5.73E-04	0.82	0.79	0.03
**cg24744258**	chr17:40939380	WNK4	Body	II	2.10E-04	1	2.13E-04	0.65	0.74	-0.09
**cg00363312**	chr7:139333296	HIPK2	Body	II	2.16E-04	1	4.58E-04	0.84	0.79	0.05
**ch.16.725323R**	chr16:23535908	EARS2	Body	II	2.20E-04	1	2.01E-04	0.16	0.20	-0.04
**cg12304482**	chr15:28377846	HERC2	Body	I	2.27E-04	1	2.67E-04	0.90	0.87	0.03
**cg13917151**	chr1:27730185	-	-	II	2.33E-04	1	5.55E-04	0.69	0.65	0.04
**cg02997111**	chr19:41698906	CYP2S1	TSS1500	II	2.38E-04	1	5.22E-05	0.08	0.10	-0.02
**cg17089179**	chr2:42990647	OXER1	1stExon	I	2.39E-04	1	8.39E-05	0.92	0.87	0.05
**cg02288001**	chr5:111496931	NCRNA00219	Body	II	2.39E-04	1	6.38E-04	0.12	0.14	-0.02
**cg26020521**	chr11:8680436	TRIM66	TSS200	II	2.47E-04	1	5.37E-04	0.16	0.22	-0.05
**cg27033923**	chr15:41106683	ZFYVE19	3'UTR	II	2.55E-04	1	5.42E-04	0.89	0.87	0.03
**cg01169396**	chr5:179372900	-	-	II	2.61E-04	1	4.14E-04	0.78	0.83	-0.05

**cg07128326**	chr7:973490	ADAP1	Body	II	2.62E-04	1	1.44E-04	0.81	0.84	-0.02
**cg00310412**	chr15:74724918	SEMA7A	Body	II	2.68E-04	1	7.61E-04	0.16	0.20	-0.05
**cg23636406**	chr17:12622228	MYOCD	Body	II	2.80E-04	1	6.56E-04	0.72	0.68	0.03
**cg26999345**	chr22:37584441	C1QTNF6	TSS200	II	2.81E-04	1	5.67E-04	0.64	0.58	0.07
**cg01178486**	chr5:68664852	TAF9	Body	II	2.81E-04	1	1.17E-04	0.12	0.15	-0.02
**cg03290560**	chr3:117774686	-	-	II	3.05E-04	1	1.27E-04	0.85	0.88	-0.03
**cg05767562**	chr17:76227422	LOC283999	5'UTR	II	3.06E-04	1	8.45E-04	0.64	0.59	0.05
**cg21828899**	chr17:79980823	LRRC45	TSS1500	I	3.10E-04	1	7.55E-04	0.07	0.08	-0.01
**cg01000937**	chr14:30730636	-	-	I	3.10E-04	1	6.41E-04	0.84	0.87	-0.03
**cg01325465**	chr16:85123554	KIAA0513	3'UTR	II	3.16E-04	1	7.66E-04	0.19	0.23	-0.05
**cg10930101**	chr17:62085466	ICAM2	TSS1500	II	3.18E-04	1	6.17E-04	0.87	0.85	0.02
**cg11070176**	chr11:70489806	SHANK2	Body	II	3.18E-04	1	2.46E-04	0.68	0.61	0.07
**cg17170741**	chr17:17942558	ATPAF2	TSS200	I	3.19E-04	1	6.20E-04	0.09	0.10	-0.01
**cg14374521**	chr10:124638874	FAM24B	5'UTR	I	3.21E-04	1	2.26E-04	0.24	0.16	0.08
**cg21041329**	chr8:56686126	TMEM68	TSS1500	I	3.22E-04	1	5.45E-04	0.09	0.10	-0.01
**cg16818361**	chr3:118792317	IGSF11	Body	II	3.24E-04	1	3.89E-04	0.90	0.88	0.02
**cg15589582**	chr3:5261136	EDEM1	3'UTR	II	3.25E-04	1	5.54E-04	0.41	0.50	-0.09

[1]Guintivano, J., Aryee, M. J. & Kaminsky, Z. A. *Epigenetics* 8, 290-302,(2013).

SUPPLEMENTARY TABLE 6 - List of differentially methylated regions (FDR<0.05) in the prefrontal cortex. Regions are annotated with genomic position (hg19, GRCh37), nearest gene and number of probes.

Genomic Region	Gene	Probes	Beta Difference	Mean SZ	Mean control	p-value	FDR
Gene body							
**chr12:101342113-101480470**	ANO4	8	0.02	0.65	0.63	3.92E-06	3.07E-02
**chr6:5999266-6006917**	NRN1	29	-0.02	0.24	0.26	8.28E-06	3.07E-02
**chr8:38125449-38126556**	PPAPDC1B	5	-0.01	0.16	0.17	2.65E-06	3.07E-02
**chr9:130830948-130868874**	NAIF1	9	-0.03	0.39	0.42	5.10E-06	3.07E-02
**chr19:36266840-36269387**	SNX26	2	-0.03	0.44	0.47	6.85E-06	3.07E-02
**chr14:77296234-77335130**	C14orf166B	6	0.02	0.68	0.66	1.23E-05	3.25E-02
**chr19:6414982-6424217**	KHSRP	7	-0.02	0.58	0.61	1.06E-05	3.25E-02
**chr14:24550845-24551147**	NRL	3	-0.02	0.23	0.25	1.40E-05	3.26E-02
**chr15:85361858-85383124**	ALPK3	6	0.01	0.74	0.73	1.88E-05	3.88E-02
**chr3:44397397-44397397**	C3orf23	1	-0.07	0.58	0.66	2.71E-05	4.19E-02
**chr8:30556362-30583924**	GSR	4	-0.02	0.71	0.73	2.49E-05	4.19E-02
**chr9:137967652-138011814**	OLFM1	24	-0.02	0.51	0.53	2.31E-05	4.19E-02
Island							
**chr10:101287381-101287846**		5	-0.02	0.16	0.18	3.61E-06	4.28E-02
**chr13:114806955-114807060**	RASA3	2	-0.07	0.62	0.69	2.80E-06	4.28E-02
**chr14:24550395-24551147**	NRL	4	-0.02	0.19	0.21	4.86E-06	4.28E-02
North Shelf							
**chr19:47204096-47204096**	PRKD2	1	-0.04	0.67	0.71	9.50E-07	9.35E-03
**chr22:29873338-29873338**		1	-0.06	0.56	0.62	1.13E-06	9.35E-03
**chr14:105235891-105236654**	AKT1	4	-0.03	0.53	0.56	2.90E-06	9.86E-03
**chr17:65037838-65037838**		1	0.04	0.78	0.74	2.97E-06	9.86E-03
**chr19:6421149-6421747**	KHSRP	2	-0.03	0.57	0.61	2.56E-06	9.86E-03
**chr7:5518071-5518918**	FBXL18	4	-0.04	0.54	0.58	1.48E-05	4.09E-02
North Shore							
**chr6:6000601-6001842**	NRN1	3	-0.04	0.66	0.7	9.90E-07	2.38E-02
**chr20:741049-741049**	C20orf54	1	0.03	0.84	0.81	4.30E-06	3.65E-02
**chr4:1201492-1202930**	LOC100130872	15	-0.03	0.54	0.57	6.08E-06	3.65E-02
**chr8:38125449-38126029**	PPAPDC1B	3	-0.02	0.21	0.24	4.59E-06	3.65E-02
**chr14:23289114-23290798**	SLC7A7	5	0.02	0.73	0.72	1.56E-05	4.16E-02
**chr15:42839559-42840397**	LRRC57	2	-0.02	0.45	0.46	1.29E-05	4.16E-02
**chr2:237079122-237079967**		5	-0.03	0.28	0.31	1.53E-05	4.16E-02
**chr8:30583924-30583924**	GSR	1	-0.06	0.62	0.68	1.18E-05	4.16E-02
**chr9:130964374-130964374**	CIZ1	1	-0.04	0.61	0.65	1.45E-05	4.16E-02

South Shelf							
**chr14:77503345-77503345**		1	-0.04	0.64	0.68	3.12E-06	4.86E-02
TSS1500							
**chr17:46692534-46693336**	HOXB8	4	-0.02	0.24	0.27	2.20E-06	4.41E-02
TSS200							
**chr4:2627014-2627142**	FAM193A	4	-0.04	0.5	0.53	1.17E-07	2.02E-03
UTR'3							
**chr14:105235891-105236654**	AKT1	4	-0.03	0.53	0.56	2.91E-06	1.17E-02
**chr1:57383752-57383752**	C8A	1	0.03	0.82	0.79	2.18E-06	1.17E-02
**chr1:109852598-109852598**	SORT1	1	-0.04	0.59	0.64	1.37E-06	1.17E-02
**chr20:741049-741049**	C20orf54	1	0.03	0.84	0.81	5.62E-06	1.69E-02
**chr14:55819305-55819305**	FBXO34	1	-0.04	0.77	0.81	7.46E-06	1.79E-02
**chr17:695428-695661**	RNMTL1	2	-0.04	0.64	0.69	9.13E-06	1.83E-02
**chr19:55670288-55670288**	C19orf51	1	-0.04	0.72	0.76	3.66E-05	4.65E-02
**chr11:85630744-85630744**	CCDC83	1	0.05	0.69	0.64	4.25E-05	4.65E-02
**chr20:36151184-36151683**	BLCAP	3	-0.02	0.61	0.63	4.22E-05	4.65E-02
**chr14:23794739-23794739**	PABPN1	1	-0.04	0.73	0.77	3.09E-05	4.65E-02
**chr2:38970936-38970936**	SFRS7	1	-0.06	0.7	0.76	3.17E-05	4.65E-02

SUPPLEMENTARY TABLE 7 – Bisulfite-pyrosequencing primers used in validation assays.

450K probe		Gene	PCR Primers (5'-3')	Sequence analyzed (5'-3')
Probe ID	Genomic position (hg19)			
cg00565348	chr6:6003303	*NRN1*	F: ATTGTTGGTGGTTTTTAGGGATTATAT<br>R: Biot-ACACTTCCCCAACAAACCCTATCA<br>S: GTTTATTTATTTAGTAGTGTTTG	TTGAGYGTTTATTAAGGATAGAGYGTTAAGTTTGAGGTTTATTTT
cg23844013	chr1:57383752	*C8A*	F: AGGGAGAATTTATAGGATGTTAGAG<br>R: Biot-CAAAATATCTACTTACCCTACCAATTAA<br>S: GAGATAAAATAAGTAGATATTTGAA	ATAATTAAYGTTTAATAAAATAAAGTAGGATGAAAATTTTTT
cg24803255	chr13:114807060	*RASA3*	F: TTGTTTTGGTGGTGTGTGAG<br>R: Biot-ACTAACTACACCAAAACTCTAATCT<br>S: TGGTGGTGTGTGAGG	TAYGGGGTGTTTATTTGTGAGATTAGAGT

SUPPLEMENTARY TABLE 8 – Technical validation of disease associated DNA methylation at CpG sites in the *C8A, NRN1* and *RASA3* genes. *T-test values from the pyrosequencing analysis are from a one-sided test. Pyro=pyrosequencing.

	C8A (cg23844013)		***NRN1* CpG1** (cg00565348)		***NRN1* CpG2**		***RASA3*** (cg24803255)	
	450K	***Pyro***	***450K***	***Pyro***	***450K***	***Pyro***	***450K***	***Pyro***
**SZ DNA methylation (%)** (mean±sd)	82.1±1.6	82.2±1.8	35.1 ±2.2	31.6±4.1	-	32.8±4.0	56.4±4.1	66.4±3.2
**Control DNA methylation (%)** (mean±sd)	79.4±1.6	81.2±1.7	38.7±2.6	33.3±4.6	-	35.0±4.1	65.1±4.7	70.5±3.7
**Mean difference DNA methylation (%)** (SZ - Control)	2.7	0.9	-3.5	-1.9	-	-2.7	-8.7	-4.0
**T-test t-statistic**	5.61	1.84	-4.85	-1.19	-	-1.74	-6.44	-3.83
**T-test P-value***	1.67e-06	0.04	1.81e-05	0.12	-	0.04	1.03e-07	2.16e-04

SUPPLEMENTARY TABLE 9 – Details of post-mortem brain samples from Douglas Mental Health University Institute Research Centre. Schizophrenia cases and controls do not significantly differ for any of the available quality metrics (t-test p-value>0.05). SZ=schizophrenia.

	SZ	Control	Total	p-value
**Number**	18	15	33	
**Sex (male:female)**	15:3	13:2	28:5	
**Age at death (years)**	45.5 ± 16.6	42.3 ± 14.8	44.0 ± 15.7	0.28
**Total brain weight (g)**	1432 ± 188	1463 ± 175	1447 ± 179	0.32
**pH**	6.6 ± 0.28	6.48 ± 0.33	6.54 ± 0.31	0.13

SUPPLEMENTARY TABLE 10 – Details of individual post-mortem brain samples samples from Douglas Mental Health University Institute Research Centre. NA=not available.

ID	Sex	Age at death (years)	Total brain weight (g)	pH
Schizophrenia				
**MS01**	M	70	1280	6.01
**MS02**	F	73	NA	6.16
**MS03**	M	30	1321	6.42
**MS04**	F	60	1175	6.23
**MS05**	F	51	1125	6.35
**MS06**	M	50	1615	6.55
**MS07**	M	29	1520	6.27
**MS08**	M	24	NA	6.01
**MS09**	M	69	1295	6.23
**MS10**	M	38	1550	6.32
**MS11**	M	39	1680	6.16
**MS12**	M	47	1516	5.76
**MS13**	M	65	1320	6.27
**MS14**	M	26	1430	6.44
**MS15**	M	29	NA	6.52
**MS16**	M	33	1812	5.92
**MS17**	M	32	1408	6.05
**MS18**	M	54	1430	6.47
Control				
**MS19**	F	57	1250	5.73
**MS20**	M	57	1400	5.72
**MS21**	F	50	1205	6.10
**MS22**	M	43	1343	6.25
**MS23**	M	44	1375	5.41
**MS24**	M	28	1565	6.59
**MS25**	M	26	1749	6.43
**MS26**	M	30	1517	6.22
**MS27**	M	37	1491	6.21
**MS28**	M	41	1430	5.89
**MS29**	M	33	1670	6.34
**MS30**	M	66	1260	6.09
**MS31**	M	21	1783	6.27
**MS32**	M	32	1516	6.49
**MS33**	M	69	1390	6.04

SUPPLEMENTARY TABLE 11 – DNA methylation data from the Montreal dataset matching the 100 top-ranked schizophrenia-associated DMPs from the LBBND prefrontal cortex dataset. DNA methylation differences at these loci are significantly correlated across datasets (r=0.54, p=6.8e-09, see **Figure 1c**).

Probe ID	Genomic position (hg19)	Gene	Gene region	London Mean SZ	London Mean Control	London Beta difference	Montreal Mean SZ	Montreal Mean Control	Montreal Beta difference
**cg26173173**	chr8:144642813	GSDMD	Body	0.83	0.79	0.04	0.79	0.77	0.02
**cg24803255**	chr13:114807060	RASA3	Body	0.56	0.65	-0.09	0.61	0.65	-0.04
**cg00903099**	chr7:154862441	HTR5A	TSS200	0.09	0.12	-0.02	0.09	0.09	0.00
**cg08171022**	chr11:70185278	PPFIA1	Body	0.48	0.53	-0.05	0.47	0.48	-0.01
**cg02857643**	chr17:48696108	CACNA1G	Body	0.87	0.92	-0.05	0.94	0.92	0.01
**cg00236305**	chr2:1796076	MYT1L	Body	0.67	0.73	-0.06	0.69	0.71	-0.02
**cg14966346**	chr14:104152124	KLC1	Body	0.72	0.77	-0.05	0.73	0.74	-0.01
**cg13079528**	chr7:4027346	SDK1	Body	0.71	0.79	-0.08	0.81	0.83	-0.02
**cg14429765**	chr8:6492531	MCPH1	Body	0.79	0.76	0.03	0.74	0.73	0.01
**cg08602214**	chr8:22864392	RHOBTB2	Body	0.69	0.75	-0.07	0.77	0.78	-0.01
**cg19735533**	chr2:241196887			0.76	0.81	-0.06	0.77	0.76	0.02
**cg09507608**	chr22:29873338			0.56	0.62	-0.06	0.56	0.58	-0.02
**cg23844013**	chr1:57383752	C8A	3'UTR	0.82	0.79	0.03	0.78	0.79	-0.01
**cg26578910**	chr19:47204096	PRKD2	Body	0.67	0.71	-0.04	0.73	0.75	-0.02
**cg21847368**	chr13:113744010	MCF2L	Body	0.80	0.84	-0.03	0.81	0.81	0.00
**cg03607729**	chr16:25138698	LCMT1	Body	0.70	0.75	-0.04	0.69	0.69	0.00
**cg10248981**	chr10:126157948	LHPP	Body	0.55	0.63	-0.07	0.61	0.64	-0.03
**cg03445663**	chr2:242170236	HDLBP	Body	0.51	0.59	-0.08	0.60	0.63	-0.03
**cg15079231**	chr3:42572745	VIPR1	Body	0.76	0.80	-0.04	0.74	0.76	-0.02
**cg21341878**	chr4:2278462	ZFYVE28	Body	0.75	0.81	-0.05	0.80	0.81	-0.01
**cg04922803**	chr12:104444418	GLT8D2	TSS1500	0.68	0.62	0.06	0.59	0.59	0.00
**cg18857062**	chr6:43276478	CRIP3	Body	0.21	0.26	-0.04	0.23	0.25	-0.02
**cg27138871**	chr11:74950396			0.75	0.79	-0.05	0.78	0.79	-0.02
**cg27363226**	chr19:6421747	KHSRP	Body	0.62	0.67	-0.05	0.65	0.66	-0.01
**cg02187259**	chr2:16804452	FAM49A	5'UTR	0.74	0.79	-0.05	0.75	0.77	-0.02
**cg26182253**	chr13:112236782			0.60	0.67	-0.06	0.65	0.68	-0.02

**cg14370653**	chr9:137828210			0.75	0.80	-0.05	0.77	0.77	0.01
**cg14527262**	chr4:1202653	LOC100130872-SPON2	TSS200	0.56	0.63	-0.07	0.68	0.72	-0.04
**cg16393877**	chr17:65037838			0.78	0.74	0.04	0.72	0.72	0.01
**cg26130533**	chr4:1202700	LOC100130872-SPON2	TSS200	0.57	0.63	-0.06	0.58	0.58	0.00
**cg05860371**	chr4:152931291			0.63	0.69	-0.06	0.65	0.67	-0.02
**cg03363012**	chr20:741049	C20orf54	3'UTR	0.84	0.81	0.03	0.79	0.77	0.02
**cg21609813**	chr8:22004881	LGI3	3'UTR	0.77	0.73	0.04	0.75	0.73	0.02
**cg27608031**	chr16:77353824	ADAMTS18	Body	0.73	0.68	0.04	0.64	0.62	0.02
**cg21390682**	chr13:113743751	MCF2L	Body	0.68	0.73	-0.05	0.65	0.65	0.00
**cg27654476**	chr14:77503345			0.64	0.68	-0.04	0.61	0.62	-0.01
**cg20930320**	chr1:109852598	SORT1	3'UTR	0.59	0.64	-0.04	0.57	0.58	-0.01
**cg12864185**	chr1:240160972			0.17	0.22	-0.05	0.15	0.16	-0.01
**cg13119928**	chr12:53228661	KRT79	TSS1500	0.81	0.78	0.03	0.76	0.76	0.00
**cg20681783**	chr5:8839868			0.38	0.33	0.05	0.32	0.33	-0.01
**cg07114544**	chr17:9110369	NTN1	Body	0.73	0.69	0.04	0.70	0.69	0.01
**cg09419102**	chr11:65550444			0.70	0.66	0.04	0.66	0.65	0.01
**cg25778661**	chr19:56031373			0.79	0.84	-0.05	0.79	0.80	-0.01
**cg13280041**	chr6:138866865	NHSL1	Body	0.31	0.26	0.05	0.27	0.25	0.03
**cg14772639**	chr2:128711564	SAP130	Body	0.79	0.74	0.04	0.74	0.72	0.02
**cg09942424**	chr17:74913419	MGAT5B	Body	0.78	0.82	-0.04	0.79	0.79	0.00
**cg04691233**	chr11:133989435	JAM3	Body	0.84	0.80	0.04	0.77	0.77	0.01
**cg07500432**	chr18:77918588	PARD6G	Body	0.81	0.71	0.11	0.82	0.80	0.02
**cg03991188**	chr7:98870835	MYH16	TSS200	0.82	0.78	0.04	0.79	0.78	0.02
**cg21843517**	chr12:123950174	SNRNP35	Body	0.83	0.79	0.04	0.79	0.76	0.02
**cg12198176**	chr22:23415316	RTDR1	Body	0.63	0.68	-0.05	0.67	0.67	0.00
**cg19643441**	chr19:19417142	SF4	Body	0.80	0.83	-0.03	0.81	0.82	-0.01
**cg07289133**	chr16:85147146	FAM92B	TSS1500	0.74	0.69	0.05	0.66	0.66	0.00
**cg11945824**	chr6:32116963	PRRT1	3'UTR	0.58	0.64	-0.06	0.69	0.70	0.00
**cg20011075**	chr1:4008090			0.62	0.69	-0.07	0.76	0.76	0.00
**cg14752965**	chr17:17579346			0.75	0.80	-0.05	0.79	0.77	0.01
**cg07421682**	chr15:102030850	PCSK6	TSS1500	0.76	0.82	-0.05	0.80	0.80	0.00

**cg22697364**	chr5:32709859			0.26	0.22	0.04	0.16	0.17	-0.01
**cg25790133**	chr4:2627014	FAM193A	TSS200	0.51	0.57	-0.06	0.53	0.56	-0.03
**cg10622536**	chr1:36771021	C1orf113	TSS1500	0.77	0.81	-0.04	0.77	0.80	-0.03
**cg01936649**	chr6:164062746			0.76	0.82	-0.06	0.81	0.81	0.00
**cg07665929**	chr5:171523225	STK10	Body	0.59	0.66	-0.07	0.63	0.64	-0.02
**cg21375204**	chr7:73752935	CLIP2	Body	0.75	0.80	-0.05	0.86	0.88	-0.02
**cg23203234**	chr8:103751623			0.71	0.76	-0.05	0.73	0.74	-0.01
**cg04215055**	chr17:74911520	MGAT5B	Body	0.65	0.71	-0.06	0.74	0.74	0.00
**cg06168875**	chr19:13988438	NANOS3	1stExon	0.76	0.82	-0.06	0.82	0.81	0.01
**cg24079702**	chr2:106015771	FHL2	TSS200	0.17	0.21	-0.04	0.12	0.10	0.01
**cg25900150**	chr8:144601851	ZC3H3	Body	0.81	0.89	-0.07	0.90	0.91	-0.01
**cg27097992**	chr14:104800950			0.66	0.54	0.12	0.61	0.61	0.00
**cg22060153**	chr2:101889653	SNORD89	TSS200	0.68	0.72	-0.04	0.65	0.66	-0.01
**cg23996071**	chr1:60280809	HOOK1	1stExon	0.18	0.22	-0.04	0.15	0.16	-0.01
**cg07815131**	chr6:99289733			0.39	0.44	-0.06	0.44	0.44	0.00
**cg05583087**	chr3:12894947			0.46	0.51	-0.06	0.47	0.49	-0.02
**cg03724006**	chr11:62790783			0.48	0.54	-0.06	0.50	0.52	-0.03
**cg10535500**	chr6:33398610	SYNGAP1	Body	0.43	0.47	-0.04	0.38	0.41	-0.02
**cg09235936**	chr2:242170211	HDLBP	Body	0.68	0.74	-0.06	0.77	0.80	-0.03
**cg12473775**	chr11:66824650	RHOD	Body	0.72	0.68	0.04	0.66	0.66	-0.01
**cg27252696**	chr6:100912940	SIM1	TSS1500	0.07	0.09	-0.01	0.05	0.05	0.00
**cg00172872**	chr12:89461426			0.77	0.82	-0.04	0.77	0.78	-0.02
**cg07780199**	chr19:18830892	CRTC1	Body	0.73	0.77	-0.05	0.70	0.71	0.00
**cg19671331**	chr8:143509438			0.72	0.80	-0.08	0.82	0.81	0.00
**cg26925590**	chr16:139752	C16orf35	Body	0.85	0.90	-0.05	0.91	0.91	0.00
**cg02064366**	chr17:79045526	BAIAP2	Body	0.62	0.67	-0.05	0.71	0.75	-0.04
**cg17244485**	chr2:103443515			0.38	0.32	0.05	0.27	0.26	0.01
**cg23236908**	chr14:55819305	FBXO34	3'UTR	0.77	0.81	-0.04	0.77	0.78	-0.01
**cg03962217**	chr7:154796431	LOC202781	Body	0.65	0.69	-0.05	0.60	0.62	-0.02
**cg17356733**	chr21:34774627	IFNGR2	TSS1500	0.64	0.70	-0.06	0.70	0.70	0.00
**cg16040341**	chr15:83544284	HOMER2	Body	0.69	0.75	-0.05	0.72	0.73	-0.01

**cg24038762**	chr1:87817476			0.73	0.77	-0.04	0.72	0.73	-0.01
**cg05529091**	chr12:111475875	CUX2	Body	0.65	0.70	-0.05	0.64	0.68	-0.04
**cg05374733**	chr12:42853791	PRICKLE1	Body	0.80	0.83	-0.03	0.83	0.83	0.00
**cg06746318**	chr7:101755186	CUX1	Body	0.70	0.76	-0.06	0.78	0.79	-0.01
**cg08404546**	chr22:37910993	CARD10	Body	0.76	0.82	-0.05	0.78	0.80	-0.02
**cg14905600**	chr10:134226361	PWWP2B	3'UTR	0.75	0.81	-0.06	0.84	0.84	-0.01
**cg10976478**	chr2:241263838			0.84	0.80	0.04	0.79	0.79	0.00
**cg21399807**	chr2:54955581	EML6	Body	0.74	0.78	-0.04	0.69	0.70	-0.01
**cg21776682**	chr8:1387801			0.68	0.73	-0.05	0.68	0.69	-0.02
**cg26593722**	chr16:87376314	FBXO31	Body	0.80	0.85	-0.05	0.90	0.91	-0.01
**cg04543233**	chr20:21689105	PAX1	Body	0.16	0.19	-0.04	0.14	0.15	-0.01
**cg06614697**	chr1:3696285			0.62	0.67	-0.05	0.64	0.66	-0.02

SUPPLEMENTARY TABLE 12 - Top 20 enriched GO terms for genes in the WGCNA modules most highly associated with schizophrenia ('black' and 'pink'). GO enrichment for biological processes was performed using EASE (DAVID). Table shows GO term, number of genes (count), and p-values for EASE score and Benjamini-Hochberg adjustment.

Gene Ontology Term	GO:ID	Count	p-value	FDR Value
Black module				
neuron projection	GO:0043005	224	2.09E-14	1.32E-10
neuron part	GO:0097458	247	1.24E-11	3.92E-08
dendrite	GO:0030425	124	1.97E-11	4.02E-08
synapse	GO:0045202	170	2.99E-11	4.02E-08
nervous system development	GO:0007399	453	3.19E-11	4.02E-08
cytoskeletal protein binding	GO:0008092	207	2.89E-10	3.03E-07
cell projection	GO:0042995	344	7.85E-10	7.06E-07
synapse part	GO:0044456	126	5.42E-09	4.27E-06
generation of neurons	GO:0048699	303	6.26E-09	4.39E-06
transmission of nerve impulse	GO:0019226	184	8.17E-09	5.15E-06
multicellular organismal signaling	GO:0035637	188	1.37E-08	7.87E-06
neuron projection morphogenesis	GO:0048812	153	1.50E-08	7.89E-06
neuron projection development	GO:0031175	175	1.93E-08	9.37E-06
neurogenesis	GO:0022008	313	3.07E-08	1.38E-05
synaptic transmission	GO:0007268	176	3.50E-08	1.47E-05
neuron development	GO:0048666	200	5.10E-08	2.01E-05
cell junction	GO:0030054	213	6.27E-08	2.32E-05
cell morphogenesis involved in neuron differentiation	GO:0048667	145	1.51E-07	5.28E-05
synaptic membrane	GO:0097060	81	2.60E-07	8.63E-05
axon development	GO:0061564	139	4.88E-07	1.54E-04
Pink module				
cell junction	GO:0030054	161	2.58E-10	1.54E-06
nervous system development	GO:0007399	302	4.11E-09	1.23E-05
homophilic cell adhesion	GO:0007156	47	7.42E-09	1.48E-05
synapse	GO:0045202	110	5.87E-08	8.56E-05
neuron projection development	GO:0031175	122	7.18E-08	8.56E-05
neurogenesis	GO:0022008	213	1.95E-07	1.83E-04
calcium ion binding	GO:0005509	129	2.14E-07	1.83E-04
generation of neurons	GO:0048699	202	3.04E-07	1.95E-04
neuron projection morphogenesis	GO:0048812	104	3.19E-07	1.95E-04

neuron projection	GO:0043005	132	3.26E-07	1.95E-04
neuron development	GO:0048666	137	3.72E-07	2.01E-04
neuron differentiation	GO:0030182	160	5.70E-07	2.83E-04
cell development	GO:0048468	220	8.21E-07	3.77E-04
dendrite	GO:0030425	75	1.07E-06	4.55E-04
system development	GO:0048731	488	1.89E-06	7.50E-04
synaptic membrane	GO:0097060	56	2.58E-06	8.62E-04
cell morphogenesis involved in neuron differentiation	GO:0048667	98	2.60E-06	8.62E-04
synapse part	GO:0044456	81	2.60E-06	8.62E-04
transmission of nerve impulse	GO:0019226	119	2.86E-06	8.97E-04

SUPPLEMENTARY TABLE 13 - Correlation between DNA methylation and brain development (days post conception) in a cohort of 179 human fetal brain samples, for the top 100 ranked DMPs between SZ and controls in prefrontal cortex. DMP=differentially methylated probe.

SZ DMP rank	Probe ID	Gene name	Pearson's R	p-value	FDR
74	cg03724006		-0.62	1.31E-20	1.28E-18
62	cg07665929	STK10	-0.58	2.44E-17	1.20E-15
6	cg00236305	MYT1L	-0.54	4.24E-15	1.39E-13
59	cg25790133	FAM193A	-0.53	1.40E-14	3.43E-13
69	cg27097992		0.53	2.49E-14	4.88E-13
86	cg03962217	LOC202781	-0.51	3.39E-13	5.54E-12
41	cg07114544	NTN1	0.45	2.18E-10	3.05E-09
87	cg17356733	IFNGR2	-0.44	9.63E-10	1.18E-08
53	cg07289133	FAM92B	0.43	2.33E-09	2.51E-08
77	cg12473775	RHOD	0.43	2.56E-09	2.51E-08
89	cg24038762		0.41	1.70E-08	1.36E-07
93	cg08404546	CARD10	-0.41	1.80E-08	1.36E-07
100	cg06614697		-0.41	1.67E-08	1.36E-07
4	cg08171022	PPFIA1	-0.4	2.33E-08	1.63E-07
92	cg06746318	CUX1	0.4	2.68E-08	1.75E-07
8	cg13079528	SDK1	0.36	6.13E-07	3.75E-06
95	cg10976478		0.36	6.70E-07	3.86E-06
31	cg05860371		-0.35	1.40E-06	7.53E-06
75	cg10535500	SYNGAP1	-0.35	1.46E-06	7.53E-06
21	cg04922803	GLT8D2	-0.35	1.71E-06	8.38E-06
67	cg24079702	FHL2	0.34	3.93E-06	1.83E-05
83	cg02064366	BAIAP2	-0.32	9.07E-06	4.04E-05
12	cg09507608		-0.3	4.49E-05	1.91E-04
90	cg05529091	CUX2	-0.29	7.18E-05	2.93E-04
22	cg18857062	CRIP3	-0.28	1.65E-04	6.47E-04
49	cg03991188	MYH16	-0.27	2.39E-04	9.01E-04
97	cg21776682		0.26	3.40E-04	1.23E-03
35	cg21390682	MCF2L	0.25	6.90E-04	2.42E-03
47	cg04691233	JAM3	0.24	1.08E-03	3.65E-03
48	cg07500432	PARD6G	0.24	1.15E-03	3.76E-03
9	cg14429765	MCPH1	0.23	1.90E-03	6.01E-03
57	cg07421682	PCSK6	0.22	3.27E-03	1.00E-02
85	cg23236908	FBXO34	0.22	3.65E-03	1.08E-02
81	cg19671331		-0.2	6.06E-03	1.75E-02
26	cg26182253		-0.2	6.45E-03	1.81E-02
63	cg21375204	CLIP2	0.2	8.15E-03	2.22E-02
66	cg06168875	NANOS3	0.19	1.10E-02	2.91E-02
61	cg01936649		0.19	1.15E-02	2.97E-02

15	cg21847368	MCF2L	0.18	1.47E-02	3.69E-02
51	cg12198176	RTDR1	0.18	1.63E-02	3.99E-02
29	cg16393877		0.17	2.01E-02	4.76E-02
72	cg07815131		-0.17	2.07E-02	4.76E-02
79	cg00172872		-0.17	2.09E-02	4.76E-02
24	cg27363226	KHSRP	-0.17	2.24E-02	4.99E-02
7	cg14966346	KLC1	0.17	2.61E-02	5.44E-02
46	cg09942424	MGAT5B	0.17	2.56E-02	5.44E-02
88	cg16040341	HOMER2	0.17	2.58E-02	5.44E-02
76	cg09235936	HDLBP	0.16	2.88E-02	5.88E-02
28	cg14527262	LOC100130872-SPON2	0.16	3.26E-02	6.52E-02
20	cg21341878	ZFYVE28	0.15	4.02E-02	7.88E-02
13	cg23844013	C8A	0.15	4.22E-02	8.11E-02
37	cg20930320	SORT1	-0.15	4.53E-02	8.54E-02
18	cg03445663	HDLBP	0.14	5.52E-02	1.02E-01
55	cg20011075		0.14	6.32E-02	1.15E-01
64	cg23203234		0.13	8.72E-02	1.55E-01
96	cg21399807	EML6	0.13	9.39E-02	1.64E-01
71	cg23996071	HOOK1	-0.12	1.04E-01	1.79E-01
34	cg27608031	ADAMTS18	-0.12	1.15E-01	1.94E-01
2	cg24803255	RASA3	-0.11	1.26E-01	2.09E-01
54	cg11945824	PRRT1	-0.11	1.51E-01	2.47E-01
70	cg22060153	SNORD89	0.11	1.59E-01	2.55E-01
98	cg26593722	FBXO31	-0.1	1.63E-01	2.58E-01
33	cg21609813	LGI3	-0.1	1.72E-01	2.68E-01
91	cg05374733	PRICKLE1	0.1	1.82E-01	2.79E-01
19	cg15079231	VIPR1	0.09	2.08E-01	3.09E-01
44	cg13280041	NHSL1	-0.09	2.08E-01	3.09E-01
94	cg14905600	PWWP2B	0.09	2.11E-01	3.09E-01
14	cg26578910	PRKD2	-0.09	2.25E-01	3.24E-01
17	cg10248981	LHPP	0.09	2.31E-01	3.28E-01
99	cg04543233	PAX1	0.09	2.55E-01	3.57E-01
80	cg07780199	CRTC1	-0.08	2.60E-01	3.59E-01
27	cg14370653		0.08	2.78E-01	3.78E-01
25	cg02187259	FAM49A	0.08	3.02E-01	4.03E-01
43	cg25778661		-0.08	3.04E-01	4.03E-01
30	cg26130533	LOC100130872-SPON2	-0.07	3.41E-01	4.46E-01
73	cg05583087		0.07	3.57E-01	4.60E-01
16	cg03607729	LCMT1	-0.07	3.81E-01	4.85E-01
78	cg27252696	SIM1	0.06	3.90E-01	4.90E-01
52	cg19643441	SF4	-0.06	4.04E-01	5.01E-01
5	cg02857643	CACNA1G	0.06	4.21E-01	5.16E-01

56	cg14752965		0.05	4.74E-01	5.73E-01
39	cg13119928	KRT79	-0.05	5.01E-01	5.99E-01
10	cg08602214	RHOBTB2	-0.05	5.21E-01	6.14E-01
58	cg22697364		0.05	5.26E-01	6.14E-01
42	cg09419102		-0.04	5.69E-01	6.56E-01
3	cg00903099	HTR5A	-0.04	5.86E-01	6.62E-01
82	cg26925590	C16orf35	0.04	5.88E-01	6.62E-01
11	cg19735533		0.04	6.27E-01	6.98E-01
32	cg03363012	C20orf54	-0.03	6.70E-01	7.30E-01
36	cg27654476		0.03	6.79E-01	7.30E-01
38	cg12864185		-0.03	6.85E-01	7.30E-01
65	cg04215055	MGAT5B	-0.03	6.64E-01	7.30E-01
68	cg25900150	ZC3H3	0.03	6.97E-01	7.34E-01
23	cg27138871		0.02	8.29E-01	8.64E-01
1	cg26173173	GSDMD	0.01	8.55E-01	8.73E-01
60	cg10622536	C1orf113	0.01	8.48E-01	8.73E-01
45	cg14772639	SAP130	-0.01	8.72E-01	8.81E-01
50	cg21843517	SNRNP35	0.01	8.92E-01	8.92E-01
40	cg20681783		NA	NA	NA
84	cg17244485		NA	NA	NA
